# Phosphorylation in the dynein light intermediate chain 2 isoform enables Pin1 binding and modulates cargo-adaptor interactions

**DOI:** 10.1016/j.yjsbx.2026.100165

**Published:** 2026-09-22

**Authors:** Michael Warchol, Charles W. Kroft, Jeffrey B. Krall, Shaun Bevers, Robb Welty, Morkos A. Henen, Beat Vögeli

**Affiliations:** Department of Biochemistry and Molecular Genetics, School of Medicine, University of Colorado Anschutz Medical Campus, Aurora, CO 80045, USA

**Keywords:** Dynein light intermediate chain, Pin1, DLIC2, Motor adaptors, Phosphorylation

## Abstract

The cytoplasmic dynein motor relies on cargo-specific adaptors that bind the C-terminal domain of dynein Light Intermediate Chain (LIC-C), yet how LIC-C phosphorylation regulates adaptor engagement remains unclear. LIC-C of both LIC1 and LIC2 isoforms are largely intrinsically disordered, containing only two regions of α-helical propensity (Helix-1 and Helix-2) near their C-termini. Here, using in vitro phosphorylation and NMR-based analyses, we show that CDK1/Cyclin B extensively phosphorylates LIC-C of LIC2 (LIC2-C) without altering its overall secondary structural propensity. Phosphorylation modulates long-range intramolecular contacts, especially in the segment spanning the Helices including the interhelical region. Consequently, the engagement of cargo adaptors BICD2 but not SPDL1 or HOOK3 is altered. Phosphorylation of LIC2-C selectively enhances BICD2 contacts with Helix-2 but does not significantly change adaptor affinity. In contrast, phosphorylation enables high-affinity binding of the peptidyl-prolyl isomerase Pin1, reducing BICD2's binding affinity while leaving SPDL1 and HOOK3 largely unaffected. These findings reveal a phosphorylation-dependent, Pin1-mediated mechanism that alters the conformational ensembles sampled by LIC2, selectively tuning dynein–adaptor interactions and facilitating the cell's dynamic spatiotemporal transport requirements.

## Highlights

• LIC2-C phosphorylation reshapes long-range contacts but keeps secondary structure.
• CDK1/Cyclin B phosphorylation selectively enhances BICD2 Helix-2 contacts.
• Phosphorylation leaves SPDL1 and HOOK3 interactions largely unchanged.
• Phosphorylation enables high-affinity Pin1 binding to LIC2-C.
• Pin1 selectively weakens BICD2 binding, tuning dynein adaptor engagement.

## Graphical abstract

**Figure:**
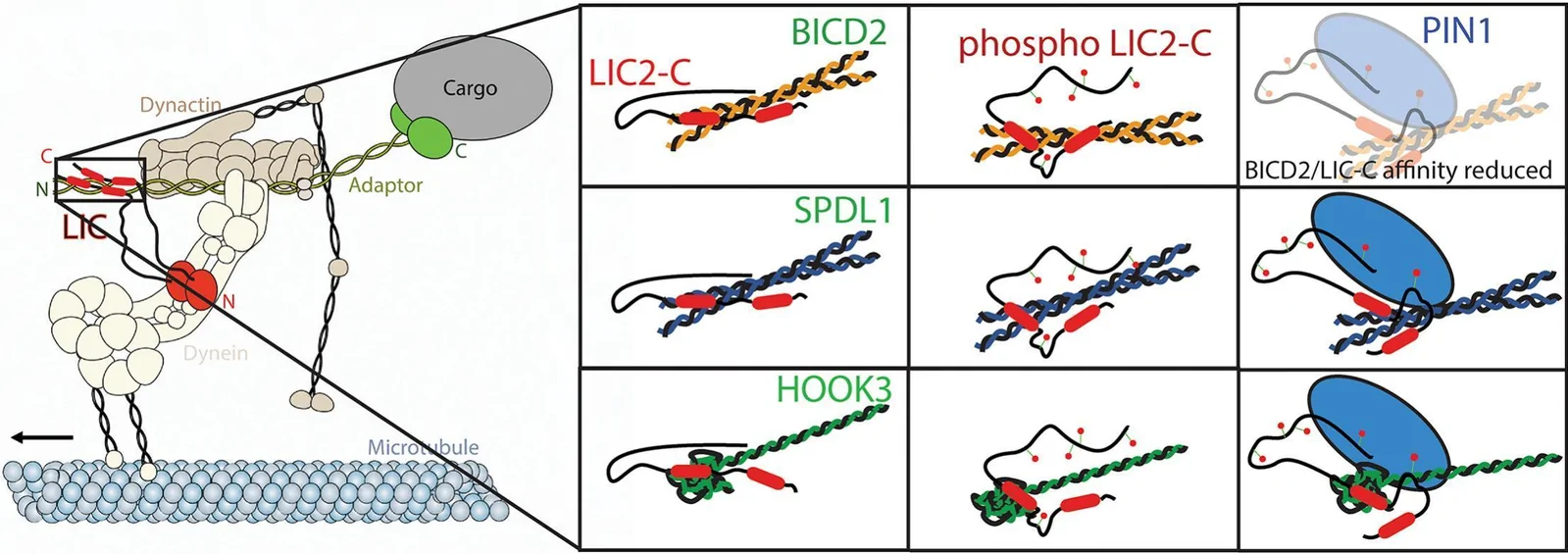

## 1 Introduction

The 1.4 MDa multi-subunit cytoplasmic dynein motor complex is one of the two classes of dynein motors that selectively facilitate retrograde intracellular cargo trafficking along microtubules via ATP-catalysis, which is critical for cellular function. Aside from cargo transport, the cytoplasmic dynein motor complex plays an essential role in mitosis, where it is found to localize to the kinetochores, centrosomes, cell cortex, nuclear envelope, and the spindle microtubules (Raaijmakers and Medema, 2014; Franker and Hoogenraad, 2013; Reck-Peterson et al., 2018). Mutations in dynein that impact axonal transport kinetics have been directly implicated in a variety of nervous system disorders such as Spinal Muscular Atrophy-Lower Extremity Dominant (SMA-LED), motor neuron disease, Perry syndrome, and Charcot-Marie-Tooth 2 disease (Franker and Hoogenraad, 2013; Lipka et al., 2013). Cytoplasmic dynein's 12-subunit complex consists of two dimerized heavy chains (HCs), which harbor the motor activity, two intermediate chains (ICs), two light intermediate chains (LICs), and six light chains (LCs). The dynein motor complex is usually accompanied by the 1.1 MDa multi-subunit cofactor dynactin, which has been shown in vitro to activate dynein-dynactin processivity in the presence of cargo-specific adaptors, or “activating adaptors” (hereafter referred to as adaptors), such as the Bicaudal-D homolog (BICD) protein 2 (BICD2) (Fig. 1A) (Reck-Peterson et al., 2018; Urnavicius et al., 2018; Urnavicius et al., 2015; Yeh et al., 2012).

**Fig. 1:**
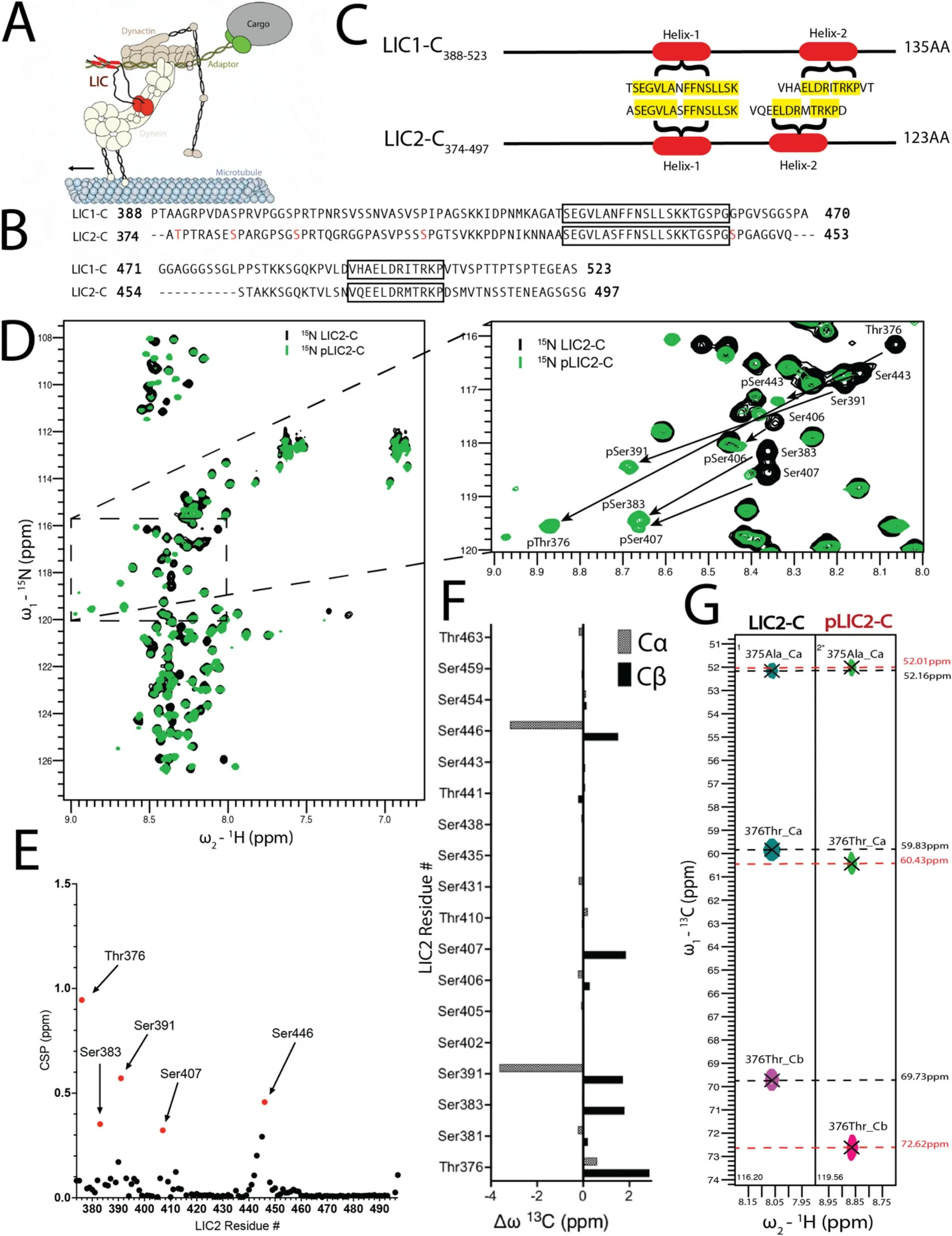
Dynein Light Intermediate Chain (LIC) and NMR chemical shift perturbations in LIC2-C induced by phosphorylation. A) Cartoon showing full-length LIC within the dynein-dynactin-adaptor complex. The N-terminal red ovals, thin black lines and thick red lines designate the GTPase-like domains, the disordered segments, and the transiently formed helices of LIC. Note that dynein is in dimeric state. B) Sequence alignment of LIC1-C_388–523_ and LIC2-C_374–497_ with residues significantly phosphorylated by Cdk1/Cyclin B highlighted in red and the transient α-helices in black boxes. C) Domain diagram of LIC1-C and LIC2-C constructs with helices shown as red ovals and conserved sequences in helices highlighted yellow. D) Overlay of ^1^H–^15^N HSQCs for unphosphorylated LIC-2 (black) and phosphorylated LIC2-C (green) and extension of region showing downfield CSPs. E) Chemical shift perturbations (ppm) plotted against LIC2-C residues after phosphorylation. Marked red are the five residues confirmed to be highly phosphorylated by NMR and mass spectrometry. F) LIC2-C and pLIC2-C ΔCα and ΔCβ chemical shift differences between phosphorylated and unphosphorylated LIC2 of residues that experienced the most significant shifts in the ^1^H–^15^N HSQC spectra. G) Backbone Cα and Cβ assignments for T376 in LIC2-C and pLIC2-C showing chemical shift changes in the ^13^C dimension after phosphorylation. (For interpretation of the references to colour in this figure legend, the reader is referred to the web version of this article.)

In addition to BICD2 and its family, several other proteins are or are likely to be adaptors, including CCDC88B, FIP3, HAP1, HOOK1–3, JIP3, Ninein (NIN) and Ninein-like, NuMa, RILP, SPDL1 (Spindly), and TRAK1 (Celestino et al., 2019; Gama et al., 2017; Lee et al., 2020; Lee et al., 2018a; Renna et al., 2020; Schroeder et al., 2014; Henen et al., 2021). The ability for dynein to bind and be activated by a wide variety of adaptors may explain the lack of diversity in cytoplasmic dynein isoforms and how a single motor can facilitate the transport of many different cargos. Adaptors share several structural and functional features that allow for interactions with LIC and cargo recruitment, such as a propensity to form long coiled-coils and LIC-interacting sequences on adaptors containing either a CC1 box, a HOOK domain, a RH1 domain, or EF-hand pairs (d'Amico et al., 2022; Yildiz and Zhao, 2023; Vilela et al., 2019; d'Amico et al., 2026). Structural characterization of the interactions between several adaptors and the cytoplasmic dynein LIC1 isoform revealed that the first helical segment (Helix-1) of the intrinsically disordered dynein subunit LIC1 C-terminus (LIC1-C) constitutes the main interaction site for the adaptors BICD2, Spindly (SPDL1), and HOOK homolog 3 (HOOK3) with *K*_D_ values ranging from ∼1 to 6 μM (Celestino et al., 2019).

Vertebrates possess two closely related LIC isoforms (LIC1 and LIC2) that also can occupy mutually exclusive dynein complexes and have partially orthogonal functional roles (Even et al., 2019; Tynan et al., 2000). While siRNA depletion of LIC2 in HeLa cells led to drastic spindle mis-orientation and prolonged metaphase arrest in the presence of an active Spindle Assembly Complex (SAC), LIC1 depletion showed minimal spindle mis-orientation but is reported to be required for metaphase to anaphase progression (Mahale et al., 2016a; Mahale et al., 2016b). Other unique phenotypic consequences of LIC2 depletion were preferential transport of NuMA to one spindle pole from the cell cortex, exclusive engagement of Par3, regulation of spindle length, congression of chromosomes to the equatorial plane, and being responsible for the removal of SAC attachment sensors (Mahale et al., 2016a; Mahale et al., 2016b). The distinct roles of the two isoforms remain largely undefined; however, immunohistochemical examination of human colorectal tissue samples and analysis of a colorectal cancer dataset revealed upregulation of LIC1 in tumors, whereas LIC2 was unchanged, suggesting significant differences do exist (Even et al., 2019).

NMR spectroscopic analysis of backbone chemical shifts of LIC1-C indicates a predominance for structural disorder with the presence of two regions exhibiting α-helical propensity termed Helix-1 and Helix-2 (Celestino et al., 2019). Pulldown assays and NMR spectroscopy data of LIC1 revealed that, in addition to making significant contacts with Helix-1 and fewer contacts with Helix-2, adaptors contact with residues N-terminal to Helix-1 (Celestino et al., 2019). Mutations of two leucine residues in Helix-1 of LIC1-C and LIC2-C abrogated binding for both constructs with six adaptors tested (BICD2, SPDL1, HOOK3, RILP1, FIP3, and NIN) (Celestino et al., 2019). With a 65.7% sequence homology between LIC1 (DC1L1) and LIC2 (DC1L2), it is unsurprising that LIC2 was also shown to be mostly structurally disordered with the exception of two regions exhibiting α-helical propensity (residues 423–439, and 468–477) also termed Helix-1 and Helix-2 (Henen et al., 2023). When comparing LIC2-C to LIC1-C, it is interesting to note that the linker between the helices is longer in LIC1 than it is in LIC2 (Fig. 1B and C) and that there is sequence conservation in both helices between isoforms. Interactions between the LIC2-C and adaptors have not been characterized on a structural level.

Dynein LIC is rich in post-translational modification (PTM) sites, which can dramatically expand the spectrum of different dynein complexes beyond the options provided by the different adaptors and may even compensate for the lack of diversity in dynein isoforms (Kumari et al., 2021a). A recent study identified four conserved Cdk1/Cyclin-B phosphorylation sites in LIC1: S207 in the N-terminal domain remains phosphorylated through mitosis, whereas S398, S405, and T408 in the C-terminal domain (LIC1-CTD) are phosphorylated during mitosis (Kumari et al., 2021b). Dephosphorylation of these phospho-sites in cell models was shown to promote prophase centrosome-nuclear envelope detachment, impairing metaphase chromosome congression, and mitotic Golgi fragmentation. Conformational and functional regulations achieved through PTMs can be further coordinated through the Peptidyl-prolyl cis-trans isomerase Pin1 (prolyl isomerase interacting NIMA 1) by binding phosphorylated serine-proline/threonine-proline (SP/TP) sites and isomerizing the adjacent prolines (K Ping et al., 1996). Pin1 is a key cell cycle regulator required for progression through mitosis via interactions with phosphorylated SP/TP motifs present in mitosis-specific phosphoproteins (Schutkowski et al., 1998). In fact, Dynein LIC1 was pulled down by Pin1 from mitotic lysates, and treatment with λ-phosphatase resulted in loss of Pin1-LIC1 binding (Kumari et al., 2021b). In vitro binding assays further identified the Pin1 binding site as the LIC1-C, suggesting potential implications in adaptor regulation (Celestino et al., 2019).

In light of previously published data on LIC1-C, we aimed to characterize LIC2-C at the molecular level and to examine the role of phosphorylation in its interaction with Pin1 and various adaptors. Here, we propose multiple phosphorylation sites in the LIC2 C-terminal domain (LIC2-C_374–497_), as evidenced by orthogonal analytical methods such as NMR and Mass spectrometry. With support from our previous NMR backbone assignment of the LIC2-C, we show that the adaptors BICD2, HOOK3, and SPDL1 primarily interact with Helix-1 of LIC2-C but also produces enhanced NMR signal broadening in Helix-2 and to a lesser degree residues preceding Helix-1, suggesting interactions with both helices. We reveal that interactions of adaptors with in vitro phosphorylated LIC2-C (pLIC2-C) result in additional signal broadening. Lastly, we show that the peptidyl-prolyl isomerase Pin1 tightly binds to pLIC2-C with a submicromolar apparent affinity but not to unphosphorylated LIC2-C, resulting in distinct adaptor binding affinities. Our investigation of the Pin1-pLIC2-C complex at the molecular level reveals a potential regulatory role that modulates LIC2-C and adaptor interactions.

## 2 Results

### 2.1 Cdk1/Cyclin B highly phosphorylates LIC2-C

To show that the dynein LIC2 C-terminus (LIC2-C) containing residues 374–497 (Fig. 1B) is phosphorylated and to identify phospho-sites, we in vitro phosphorylated ^15^N (Schroeder et al., 2014),C-labeled LIC2-C with the human mitotic kinase Cdk1/Cyclin-B and turned to NMR spectroscopy. Cdk1 or Cyclin-dependent kinase 1 is a type of serine/threonine kinase that forms a complex with Cyclin B to phosphorylate specific proteins. A near-complete backbone assignment of LIC2-C at 840 μM was previously performed by Henen et al., providing us with a ^1^H–^15^N correlation spectrum of the non-phosphorylated LIC2-C construct (Supplemental Fig. 1A) (Henen et al., 2023).

After the phosphorylation reaction, we acquired a ^1^H–^15^N heteronuclear single-quantum coherence (HSQC) spectrum of phosphorylated LIC2-C (pLIC2-C) at 80 μM, producing a 2D fingerprint correlation spectrum (Fig. 1D) (Cavanagh, 1996). Backbone assignment of pLIC2-C was accomplished using 3D (H)NCαCβ, HNN, HNCO, and (H)CC(*CO*)NH experiments at 25 °C (Supplemental Fig. 1B). An overlay of the ^1^H–^15^N spectra of LIC2-C and pLIC2-C revealed significant ^1^H-downfield chemical shifts for T376, S383, S391, S407, S443, and S446. We also observed notable chemical shifts for non-serine/threonine residues A375, G442, G445 and several others in the pLIC2-C spectrum, likely due to their proximity to phosphorylated residues and/or other changes in the local chemical environment.

The largest chemical shift perturbations (CSPs) in pLIC2-C are from serine and threonine residues in the N-terminal region and the linker between transient Helix-1 and Helix-2 but also residues proximal to serines or threonines (Fig. 1E). In the linker region, S446 experiences the greatest CSP, and subsequent residues N-terminal and C-terminal to S446 experience gradually reducing CSPs. In most cases, chemical shift effects are not expected to extend beyond 2–3 residues on either side of the modified residue in random-coil states; however, structural rearrangements or changes in the conformational ensembles of IDPs can allow them to have long-range effects (Hendus-Altenburger et al., 2017).

NMR chemical shift measurements of unmodified and modified amino acids reveal that, for most of the random coil peptides, addition of a PTM made little to no difference to the backbone chemical shifts of the residue (NH, HN, Hα, Cα, and CO). In contrast, phosphates in structured regions might allow for interactions with the backbone HN of other residues nearby in space (Conibear et al., 2019). Conibear et al. calculated the chemical shifts of modified and unmodified residues in random coils; they found that while phosphorylation in serine results in Cα shift changes from 58.6 to 57.6 ppm and Cβ shift changes from 63.8 to 66.7 ppm, in threonine it alters Cα shifts from 62.0 to 61.9 ppm and Cβ shifts from 69.8 to 73.9 ppm (Conibear et al., 2019). Using the previous LIC2-C and pLIC2-C backbone assignments, we calculated the Cα and Cβ chemical shift differences between the phosphorylated and unphosphorylated residues (Fig. 1F). These shifts were most evident in the 3D spectrum of T376, which experiences a significant down-field shift in its Cβ after phosphorylation as well as a minor up-field shift of the Cα of A375 (Fig. 1G). Overall, given the observed ΔCα and ΔCβ chemical shift values, we conclude that T376, S383, S391, S407, and S446 are phosphorylated in pLIC2-C, in agreement with the largest CSPs from the HSQC data. On the other hand, S443 which also undergoes significant CSP in the HSQC spectrum, is not phosphorylated but is impacted by the phosphorylation of the nearby S446. A comparison of the shifted peaks for phosphorylated residues and the residual peaks that were assigned in the non-phosphorylated LIC2-C sample allowed us to calculate the degree of phosphorylation per residue and revealed that S407 was the least phosphorylated at 72%, while S446 was completely phosphorylated (Table 1).

**Table 1:** Degree of phosphorylation calculated by peak height intensities. * Phosphorylation efficiency of each amino acid calculated by comparing the peak heights between the weaker remaining unphosphorylated peak and the new shifted phosphorylated peak of the same residue. Uncertainties were calculated by propagation of spectral noise (6.99 × 10^8^ and 1.03 × 10^9^ for phosphorylated and unphosphorylated sample, respectively).

LIC2-C residue	Phosphorylated peak height	Non-phosphorylated peak height	Non-phospho/Phospho peak height ratio	% Phosphorylated*
376Thr	1.63 × 10^11^	9.97 × 10^9^	0.0612 ± 0.0063	94 ± 0.63
383Ser	1.36 × 10^11^	6.72 × 10^9^	0.0496 ± 0.0076	95 ± 0.76
391Ser	8.02 × 10^10^	1.38 × 10^10^	0.172 ± 0.013	83 ± 1.3
407Ser	3.75 × 10^10^	1.03 × 10^10^	0.275 ± 0.028	72 ± 2.8
446Ser	4.80 × 10^10^	−2.05 × 10^8^	−0.004 ± 0.022	100–1.8

By applying a phosphoproteomics approach using bottom-up Liquid-chromatography mass spectrometry (LC-MS) on pLIC2-C (+Cdk1/Cyclin-B) and a control sample (-Cdk1/Cyclin B) we assessed site-specific phosphorylation in a manner orthogonal to NMR. Using the protease GluC to produce peptides, our LC-MS fragmentation analysis revealed a total of 15 serine/threonine phosphorylation sites (Supplemental Fig. 2A), which contain those identified by NMR with the exception of pT376 and pS407. While our NMR results suggest that T376 and S407 are phosphorylated, we observed no indication of this in our LC-MS results likely because of reduced GluC protease accessibility due to steric hinderance of the cleavage site by a phosphate or local rearrangement after phosphorylation. We observed a relative abundance value of 312,800.2 for the peptide containing T376 (NH2-WATPTRASE-COOH) in the control sample and of 10,144.6 in the pLIC2-C sample in comparison to the control LIC2-C sample, indicating that indeed the cleavage efficiency for this peptide decreased after phosphorylation. Furthermore, fragmentation analysis of S407-containing peptides may yield lower confidence due to the presence of three successive serines (S405-S407) which are flanked by prolines, though most peptides produced reliable fragmentation patterns and were confidently assigned (Supplemental Fig. 2C).

Despite taking a qualitative approach with our LC-MS analysis, we calculated the Log2 fold-changes (Log2FC) (Supplemental Fig. 2B) in peptide intensities of phosphorylated sites in the +Cdk1/Cyclin-B condition relative to the -Cdk1/Cyclin-B condition. A Log2FC value >1 indicates higher phosphorylation in the experimental condition, <1 indicates lower phosphorylation, and = 1 indicates no change in phosphorylation status. The only significant Log2FC values were those of phosphorylated S/T residues in the phosphorylated samples, almost all of which were far above a calculated value of 1 (Log2FC > 15). In addition, S383, which also showed a high intensity in the phosphorylated sample, has a similar value in the control, resulting in a Log2FC value of 0.803. Therefore, the data suggests phosphorylation of this residue despite a Log2FC < 1, in agreement with the NMR data. All other residues showing large CSPs upon phosphorylation rank among those with high Log2FC values, namely S383, S389, S391, and S446, confirming the results of the two orthogonal methods. While other residues show substantial Log2 fold-changes (S402, S405, T410, S431, S435, S438, S454, S459, and T463), we likely did not observe them by NMR due to their lower abundance in the total pLIC2-C population. While NMR may allow for more accurate sampling of the degree of phosphorylation and therefore the total population of LIC2 molecules being phosphorylated, LC-MS proved to be more sensitive in detecting low abundance modifications. Overall, we orthogonally characterized the site-specific phosphorylation of the dynein LIC2-C (374–497) isoform by Cdk1/Cyclin B using NMR and mass spectrometry, revealing T376, S383, S391, S407, and S446 as the primary phosphorylation sites, with additional phosphorylation throughout the LIC2-C sequence to a lesser extent. It is important to note that the NMR experiments report the average behavior of such a heterogeneous populations of in-vitro phosphorylated species.

### 2.2 Phosphorylation does not influence LIC2-C secondary structure propensity

We utilized the pLIC2-C H, N, Cα, and Cβ resonance assignments to calculate a Secondary Structure propensity (SSP) score developed by Forman-Kay and colleagues (Marsh et al., 2006). By comparing the different backbone chemical shift values to those of random coil values for each amino acid, we can assign a single residue-specific SSP score. The SSP scores vary between +1, indicating a fully formed α-helix, and − 1, indicating a fully formed β-strand. Residues in loops and intrinsically disordered regions give SSP values near 0.

Our SSP calculations for pLIC2-C show that most residues fall between 0 and − 0.2, indicating structural disorder, except for two regions, between residues 424–438 (Helix-1) and 468–477 (Helix-2), which show positive SSP scores, with a maximum of 0.33 (Fig. 2A). These moderately clustered, positively valued SSPs indicate not fully formed α-helices but a propensity to sample α-helical structures. Similarly, moderately clustered negative SSP scores indicate not fully formed β-strands but a propensity to sample them. Overall, these results align with prior SSP calculations of unphosphorylated LIC2-C and indicate that phosphorylation does not influence LIC2's secondary structure propensities (Henen et al., 2023).

**Fig. 2:**
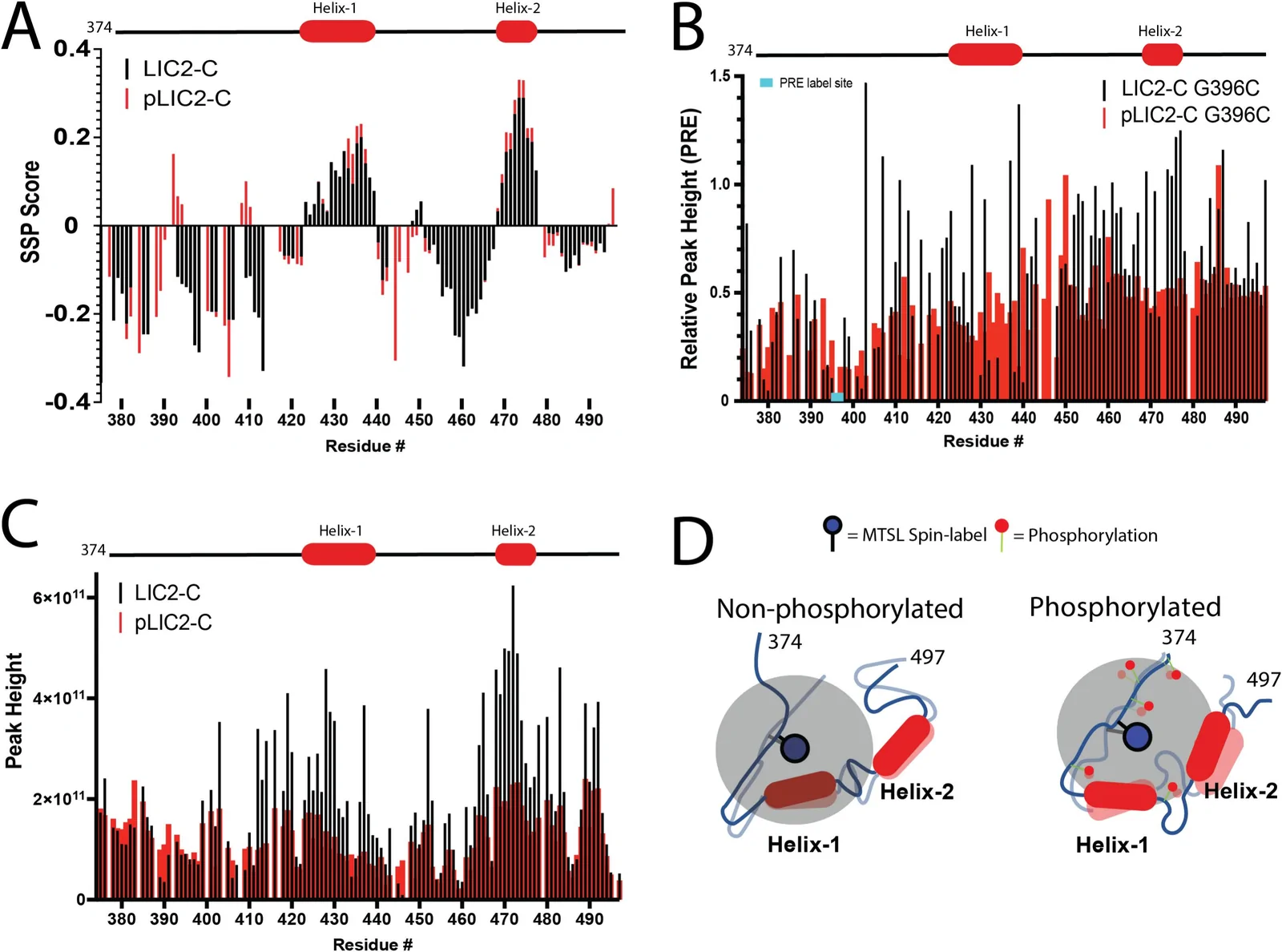
Secondary structure propensity analysis, backbone chemical shifts, and residue distance constraints for pLIC2-C. A) Secondary structure propensity calculation per residue in LIC2-C (black) and pLIC2-C (red). B) Comparison of PRE in LIC2-C and pLIC2-C. Peak intensity reductions are plotted versus residue number. C) Comparison of ^1^H–^15^N HSQC peak intensities of LIC2-C and pLIC2-C. D) A cartoon model showing LIC2-C and pLIC2-C with MTSL label (dark blue symbol; 25 Å radius, grey disc) and phosphates (red circles). A solid and a transparent copy of LIC2-C represent exchanging conformations in an ensemble of structures. The spin label on LIC2-C is closer to Helix-1 and further from Helix-2 than in pLIC2-C, while pLIC2-C exhibits more peak broadening in Helix-2 and to a lesser extent in Helix-1 than LIC2-C. (For interpretation of the references to colour in this figure legend, the reader is referred to the web version of this article.)

### 2.3 Phosphorylation favors long-range contacts of Helix-2 but reduces those with Helix-1

We have shown in the previous section that phosphorylation does not alter the secondary structure of LIC2-C. In order to gain a deeper understanding on the effect of phosphorylation on long-range interactions within LIC2-C, we applied Paramagnetic Relaxation Enhancement (PRE) to extract distance restraint information. PRE spin labels take advantage of unpaired electrons to create strong magnetic fields to enhance relaxation rates of nuclei within fixed distances (Bertini et al., 2005; Clore and Iwahara, 2009). Attaching a spin label to a known location of a protein can inform on dynamics and proximity of the spin label to other parts of the protein, as well as contacts formed with other labeled proteins, indicated by a reduction in peak heights or signal in NMR HSQCs. Using a LIC2-C G396C mutant, we first measured a standard ^1^H–^15^N HSQC to confirm that the mutation did not alter amino acid chemical environments (Supplemental Fig. 3A). After validating that the LIC2-C G396C mutant overlapped closely with the LIC2-C HSQC, we proceeded to label C396 with an MTSL (S-(1-oxyl-2,2,5,5-tetramethyl-2,5-dihydro-1H-pyrrol-3-yl)methyl) spin-label, which will enhance the relaxation rates of residues within ∼25 Å of the label. We measured an ^1^H–^15^N HSQC on the MTSL-labeled LIC2-C, and then reduced the sample with DTT before recording a second ^1^H–^15^N HSQC. By comparing the peak heights of residues in the MTSL-labeled LIC2-C and reduced LIC2-C HSQCs, we observed significantly reduced peak heights in the N-terminal region of LIC2-C localized around the MTSL spin-label as expected (Fig. 2B, Supplementary Fig. 3B). Additionally, there is reduction present around Helix-1. Slighter peak reductions were generally present throughout the entire construct. When the same PRE approach was repeated with pLIC2-C, we observed little change in the N-terminal region where the spin-label is located, but notably less peak reduction in Helix-1 and more peak reduction in Helix-2 compared to the nonphosphorylated LIC2-C (Fig. 2B, Supplementary Fig. 3C). The interhelical linker also experienced a slight reduction in peak height.

Independent support for the impact of phosphorylation on the helices came from the HSQC spectra of the wildtype LIC2-C sample (not the PRE sample) (Fig. 2C). In the non-phosphorylated case, there are large peak intensity variations along the sequence. The largest intensities were observed for Helices-1 and -2, and the regions preceding Helix-1 and following Helix-2 also exhibit larger-than-average values. On the other hand, the N-terminal ∼35 residues and the interhelical region feature lower intensities. While phosphorylation leaves the weaker peaks unchanged, Helix-2 and to a lesser extent for Helix-1 experience significant peak reduction, indicating additional conformational exchange on the μs-ms timescale or slowed down overall local tumbling caused by phosphorylation. Altered conformational populations upon phosphorylation are also present in the interhelical region as seen by the consistently different chemical shifts in that region despite of the absence of phosphorylation sites (Fig. 1E).

The overall picture that emerges from the secondary structure scores, PREs, the HSQC peak intensities, and chemical shift changes (Fig. 2D) is the following: The disordered N-terminal region is well within 25 Å of Helix-1 in nonphosphorylated LIC2-C, enabled through hinge formation allowing the N-terminal region to be in proximity of Helix-1. Phosphorylation does not disrupt or alter LIC2-C's regions of α-helical propensity. Phosphorylation adds negative charge to the N-terminal region. Upon plotting charge distribution across the sequence of LIC2-C and the pLIC2-C (Supplemental Fig. 4), reveals that the N-terminal part of Helix-1 is negatively charged as well. Potentially driven by these repulsive forces, phosphorylation loosens the hinge, resulting in a larger distance between the N-terminal segment and Helix-1. However, phosphorylation causes LIC2-C to sample overall more compact states, where the N-terminal region repositions to be closer to Helix 2 and the interhelical linker.

### 2.4 LIC2-C binds adaptors in regions of transient α-helical propensity

We previously mapped the binding sites of the cargo adaptors BICD2, HOOK3, and SPDL1 on LIC1-C using NMR spectroscopy (Celestino et al., 2019). We followed ^15^N-labeled LIC2-C by HSQC spectra upon adding increasing amounts of unlabeled adaptor segments BICD2_2–422_, HOOK3_2–239_, and SPDL1_2–359_ that are known to bind LIC (Fig. 3A) (Celestino et al., 2019). Here, we adapted these experiments to determine if LIC2-C bound these adaptors in a similar manner as LIC1-C (Fig. 3B–D). Similarly to what has been observed for LIC1-C, we observed peak disappearance during NMR titration experiments, indicative of binding. This peak disappearance can occur for several reasons, such as exchange between free and bound species or an increase in the tumbling time of the complexed molecules. Ratios of the peak intensities between LIC2-C+BICD2 and free LIC2-C (bound/free) obtained from ^1^H–^15^N HSQC titrations series with LIC2-C and the adaptor BICD2 at ratios of 1:0.5, 1:1, and 1:2 (Fig. 3B) were plotted against LIC2-C residue number to graphically display the regions involved in the interaction (Fig. 3E). The plot revealed significant decreases in peak intensity and, in some cases, peak disappearance for several residues in Helix-1 (residues 411–448) and, to a lesser extent, Helix-2 (residues 461–481).

**Fig. 3:**
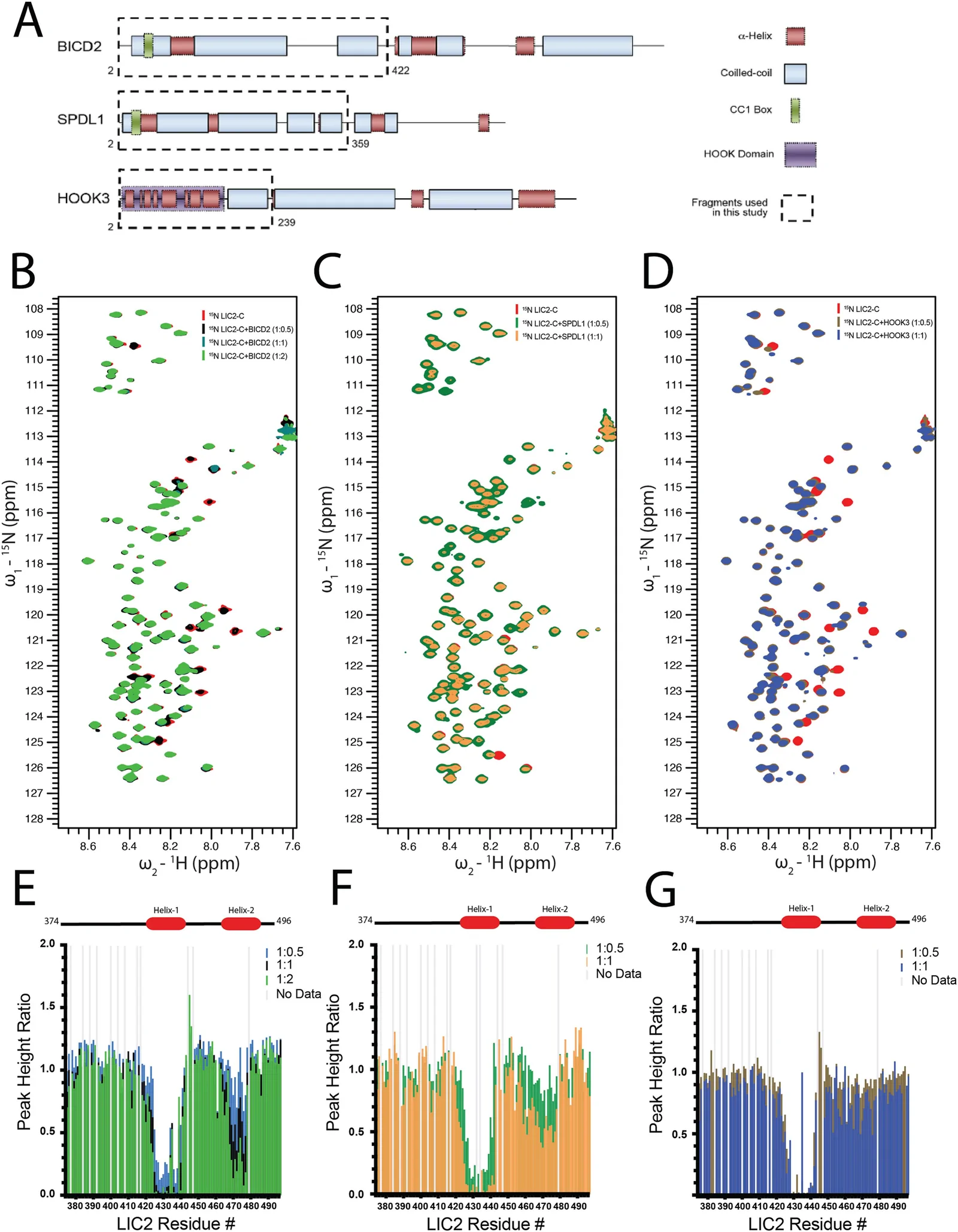
LIC2-C titration with BICD2, SPDL1, and HOOK3 probed by HSQC. A) LIC-C binding segments of BICD2, SPDL1, and HOOK3 used for binding studies. Figure adapted from reference (Celestino et al., 2019). B) ^1^H–^15^N HSQC overlays of free LIC2-C and LIC2-C+BICD2 at ratios of 1:0.5 (light blue), 1:1 (black), and 1:2 (light green). C) HSQC overlays of free LIC2-C and LIC2-C+SPDL1 at a ratio of 1:0.5 (green) and 1:1 (orange). D) HSQC overlays of free LIC2-C and LIC2-C+HOOK3 at a ratio of 1:0.5 (brown) and 1:1 (blue). The ^1^H-dimension range of the HSQC spectra in B)-D) is selected to display backbone amides but to exclude side chains. E) Peak height ratios of LIC2-C and BICD2 titrations plotted against LIC2-C residue numbers. F) Peak height ratios of LIC2-C and SPDL1 titrations plotted against LIC2-C residue numbers. G) Peak height ratios of LIC2-C and HOOK3 titrations plotted against LIC2-C residue numbers. Plots E)-G) also include grey bars to denote missing data points. (For interpretation of the references to colour in this figure legend, the reader is referred to the web version of this article.)

HSQC overlays of free LIC2-C, LIC2-C+SPDL1 at a ratio of 1:0.5, and LIC2-C+SPDL1 at a ratio of 1:1 followed a similar pattern of peak intensity decreases in Helix-1 and Helix-2. (Fig. 3F). HSQC overlays of free LIC2-C, LIC2-C+HOOK3 at a ratio of 1:0.5, and LIC2-C+HOOK3 at a ratio of 1:1 produced spectra with the complete disappearance of peaks for residues in Helix-1 but minimal or no intensity decreases in Helix-2. (Fig. 3G). In addition, we observed the emergence of new peaks for residues E426-S431, as well as L437 and S438, after HOOK3 addition (Supplemental Fig. 5A, D and G). The new peaks were slightly shifted from their original positions and were weaker in intensity than the initial peaks prior to HOOK3 addition. This difference in binding mode (Helix-1 vs. Helix-2, and peak disappearance vs. shifting) between BICD2 and SPDL1, compared to HOOK3, could be attributed to differences in their binding motifs: BICD2 and SPDL1 have an N-terminal CC1 box, whereas HOOK3 has an N-terminal HOOK domain (Lee et al., 2018b). Regardless, all adaptors tested engaged LIC2-C at Helix-1 and, to a lesser extent, with Helix-2 in the case of BICD2 and SPDL1.

### 2.5 Phosphorylation influences the degree to which adaptors contact LIC2-C Helix-2

While phosphorylation had no impact on LIC2-C secondary structure propensities, it did influence long-range aspects of the conformational ensembles, suggesting that adaptor/pLIC2-C interactions would be influenced as well. Using NMR and ^15^N-labeled pLIC2-C, we measured free pLIC2-C ^1^H–^15^N HSQC spectra and assessed changes in peak intensities upon addition of BICD2, SPDL1, and HOOK3. Due to the relatively high concentrations of Cdk1/Cyclin B-treated protein required for NMR measurements, we only conducted adaptor titrations with pLIC2-C at a ratio of 1:0.5. Free pLIC2-C spectra and pLIC2-C+BICD2, SPDL1, and HOOK3 spectra were overlayed to show peak disappearances and shift changes (Fig. 4A–C). Peak height ratios between pLIC2-C+BICD2 and free pLIC2-C plotted against the LIC2-C sequence revealed that the presence of BICD2 still retains significant contacts with Helix-1 and Helix-2 (Fig. 4D). Similarly, the pLIC2-C+SPDL1 titration showed contacts in Helix-1 with weaker contacts in Helix-2 (Fig. 4E). Titration of pLIC2-C and HOOK3 also showed conserved contacts in Helix-1, but none in Helix-2 (Fig. 4F).

**Fig. 4:**
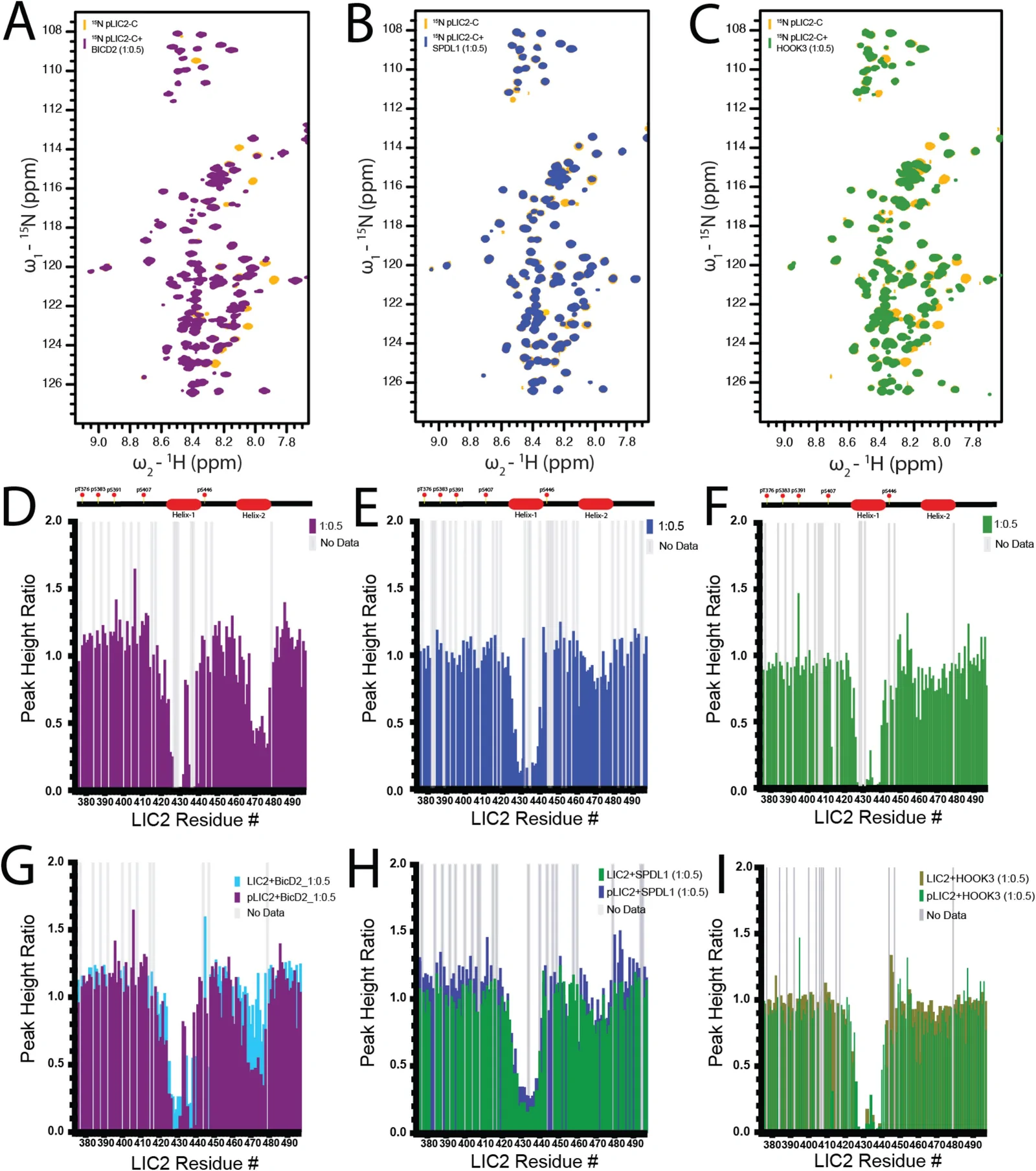
pLIC2-C titration with BICD2, SPDL1, and HOOK3 probed by HSQC and comparison to LIC2-C-adaptor titrations. A) ^1^H–^15^N HSQC overlays of pLIC2-C (yellow) and pLIC2-C+BICD2 at a ratio of 1:0.5 (purple). B) HSQC overlays of free pLIC2-C (yellow) and pLIC2-C+SPDL1 at a ratio of 1:0.5 (blue). C) HSQC overlays of free pLIC2-C (yellow) and pLIC2-C+HOOK3 at a ratio of 1:0.5 (green). The ^1^H-dimension range of the HSQC spectra in A)-C) is selected to display backbone amides but to exclude side chains. D–F) Peak height ratio plots of pLIC2-C and adaptor titrations plotted against LIC2-C residue numbers. Grey bars denote missing data points. G-I) Comparison of peak height ratios for LIC2-C+adaptors and pLIC2-C+adaptors at 1:0.5 ratio.

When comparing the peak height ratios of LIC2-C+BICD2 and pLIC2-C+BICD2 at a ratio of 1:0.5 (4G–I), we observed no change in the peak height ratios in Helix-1 but a notable reduction of peak height ratios in Helix-2, suggesting that phosphorylation allowed for more contacts to form between BICD2 and Helix-2. When we compared the peak height ratios between LIC2-C+SPDL1 and pLIC2-C+SPDL1 at a 1:0.5 ratio, we observed no change in the peak height ratios for Helix-1 or Helix-2. For LIC2-C+HOOK3 and pLIC2-C+HOOK3, we again observed no change in the peak height ratios in Helix-1 but slightly different peak height ratios in Helix-2. Overall, we have shown that all adaptors contact Helix-1 of LIC2-C and form weaker contacts with Helix-2 except for HOOK3, while phosphorylation significantly altered the binding interactions in Helix-2 for BICD2 but not the others.

Furthermore, we observed that residues E426-S431 and L437/S438, which are within the canonical adaptor binding motif (FFNXLL) in Helix-1, experienced slight peak splitting when LIC2-C was phosphorylated (Supplemental Fig. 5B, E and H). The new set of peaks was much weaker than the main peaks and varied in intensity from residue to residue.

When we added BICD2 to pLIC2-C, the main peaks would disappear while the weak minor peaks remained (Supplemental Fig. 3B). To our surprise, when HOOK3 was added to pLIC2-C, we observed the same peak splitting behavior as in the LIC2-C+HOOK3 titration experiment and the disappearance of the main peaks. However, it is difficult to determine whether phosphorylation influenced this. On the other hand, when SPDL1 was titrated into pLIC2-C samples, we observed the disappearance of the major peaks, as seen before with LIC2-C, and also the disappearance of the minor peak species that had appeared in the pLIC2-C spectrum.

### 2.6 Pin1 binds phosphorylated LIC2-C but not unphosphorylated LIC2-C

Following previous work that characterized the interaction of phosphorylated LIC1 with the peptidyl-prolyl cis/trans isomerase Pin1 using pulldown assays (Kumari et al., 2021b), we applied NMR to resolve the interaction between LIC2-C and pLIC2-C with Pin1. For a reference, we recorded a ^1^H–^15^N HSQC with 100 μM ^15^N-labeled LIC2-C. Then we added unlabeled full-length Pin 1-6xHis_1–183_ to a final concentration of 100 μM resulting in a ratio of 1:1, collected a second HSQC, and overlayed the resulting spectrum with the free LIC2-C spectrum (Fig. 5A). Using our assignment of LIC2-C, we calculated ratios of the bound LIC2-C peaks over free LIC2-C peaks (Fig. 5B) as well as CSPs of each residue upon Pin1 addition. We observed no significant differences in LIC2-C peak intensity after adding Pin1, and the greatest CSP was 0.012 ppm for S406 (Fig. 5C). Such small CSPs are within measurement uncertainty and therefore not indicative of any interaction. Overall, these results indicate that Pin1 does not bind unphosphorylated LIC2-C.

**Fig. 5:**
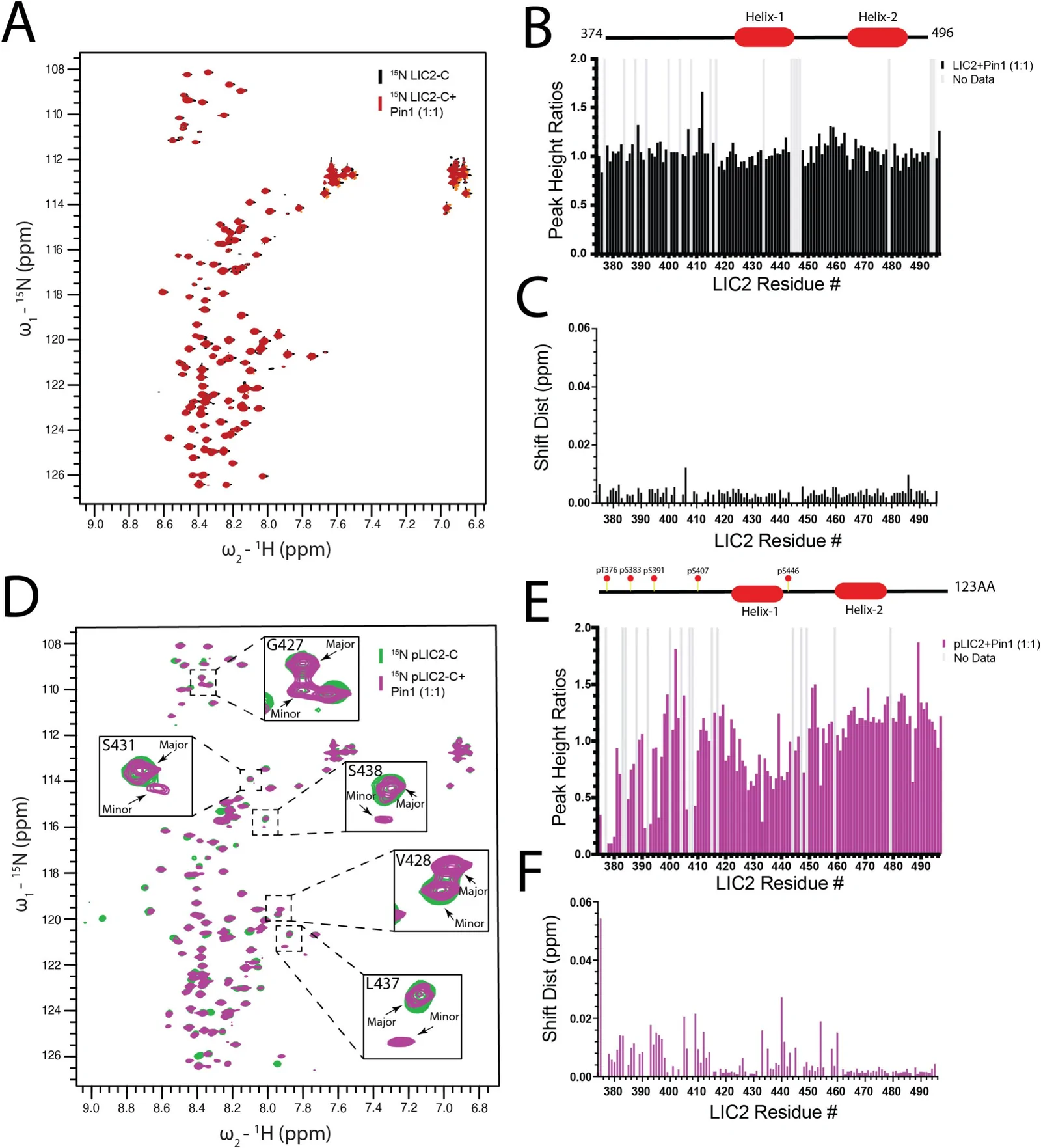
LIC2-C/pLIC2-C interactions with Pin1 probed by HSQC. A) Overlay of 100 μM ^15^N-LIC2-C (black) HSQC and 100 μM ^15^N-LIC2-C with 100 μM Pin1 (red) HSQC. B) Ratio of peak heights from LIC2-C and LIC2-C+Pin1 HSQCs (black bars) plotted against LIC2-C sequence with LIC2-C model. C) CSPs per LIC2-C amino acid plotted against the LIC2-C sequence after Pin1 addition. D) Overlay of 100 μM ^15^N-pLIC2-C (green) HSQC and ^15^N-pLIC2-C+Pin1 (purple) HSQC. E) Ratio of peak heights from pLIC2-C and pLIC2-C+Pin1 HSQCs (purple bars) plotted against LIC2-C sequence with LIC2-C model above. F) CSPs per pLIC2-C amino acid plotted against the LIC2-C sequence after Pin1 addition. (For interpretation of the references to colour in this figure legend, the reader is referred to the web version of this article.)

We proceeded to phosphorylate ^15^N-LIC2-C according to our phosphorylation protocol and acquired a HSQC spectrum of pLIC2-C at 110 μM. To validate phosphorylation and agreement with our previous assignment, we overlaid the 110 μM pLIC2-C HSQC with the ^15^N,^13^C-labeled pLIC2-C HSQC used for our initial assignment (Supplemental Fig. 6). Slight chemical shift changes of phosphorylated amino acids in ^1^H–^15^N-resolved spectra may be expected when comparing between samples due to buffer pH variation, as chemical shifts of phosphorylated residues are particularly sensitive to changes in pH. We added unlabeled Pin1 to ^15^N-pLIC2-C (1:1 stoichiometry) and recorded an HSQC spectrum. We overlayed the free pLIC2-C spectrum and pLIC2-C+Pin1 spectrum and observed several peaks disappearing, shifting, and splitting (Fig. 5D). We extracted the peak heights from the free ^15^N-pLIC2-C spectrum and compared them to those in the ^15^N-pLIC2-C+Pin1 spectrum, revealing substantial intensity differences localized to the N-terminal regions of pLIC2-C and Helix-1 (Fig. 5E).

After observing that several residues shifted in the spectra after Pin1 addition, we plotted the CSPs against the LIC2-C sequence. Significantly shifting values localized to the N-terminal region, Helix-1, and the linker. A375 undergoes the greatest CSP of 0.054 ppm, attributed to its proximity to pT376, which completely disappeared upon Pin1 addition, likely due to binding, and subsequently did not have a CSP value attributed to itself (Fig. 5F). Interestingly, the addition of Pin1 to pLIC2-C exaggerated the peak splitting of residues E426-S431, L437, and S438 to a much greater degree than in the pLIC2-C spectrum alone by producing minor peaks of near equal intensity as the major peaks. The split peaks in the pLIC2-C and pLIC2-C+Pin1 spectra directly overlap, suggesting a shared mechanism that gives rise to two distinct populations of pLIC2-C molecules on a slow-exchange time scale, making them observable by NMR-HSQC.

### 2.7 Adaptors preferentially bind to the major conformation of pLIC2-C

When Pin1 was added to pLIC2-C, many peaks disappeared; however, residues E426-S431, L437, S438, and perhaps others, which were previously split to produce a weaker intensity subpopulation of peaks in the phosphorylated pLIC2-C spectra and also upon addition of HOOK3, showed both peaks that were in some cases at near equal intensity. This suggests that Pin1 causes a population shift in pLIC2-C toward the minor population. When we then added BICD2 or HOOK3 to pLIC2-C in the presence of Pin1, we observed that the major peaks disappeared but the minor peaks remained with much greater intensity than in the pLIC2-C+ BICD2/HOOK3 samples without Pin1 (Supplemental Figs. 5C,I). This indicates that BICD2 and HOOK3 bind to the major state of pLIC2-C, effectively increasing the effective overall tumbling time and/or inducing conformation exchange. Conversely, the minor state is not bound, thus leaving the peaks unaffected. When SPDL1 was added to pLIC2-C samples containing Pin1, we observed a decrease in the major peak intensity, as well as potentially a lowering in the intensity of the minor peaks (Supplemental Fig. 5F), suggesting that SPDL1 may bind both species.

### 2.8 Pin1 binding to pLIC2-C alters the binding affinity of BICD2 and HOOK3 for pLIC2-C but not SPDL1

We assessed binding affinities with Microscale thermophoresis (MST) in which LIC2-C or pLIC2-C was labeled with a Red-Tris-NTA (nitrilotriacetic acid) dye and titrated with Pin1_2–163_, BICD2_2–422_, HOOK3_2–239_, and SPDL1_2–359_ (Table 2). NMR analysis has already revealed no interactions between non-phosphorylated LIC2-C and Pin1, and thus we did not measure an MST response curve these cases. Binding was assessed with NTA-labeled pLIC2-C and Pin1 in a single replicate, yielding a *K*_D_ of 0.88 ± 0.17 μM (Fig. 6A). We then used 50 nM LIC2-C, 50 nM pLIC2-C, and 50 nM pLIC2-C+5 μM Pin1 to determine if phosphorylation or the presence of Pin1 altered the dissociation constants for adaptors to LIC2-C. We normalized several response curves to a percentage scale so that each curve could be plotted on the same graph, as the response values differed greatly in the presence of Pin1 (Figs. 6B-D). For the BICD2 adaptor we calculated *K*_D_ values using replicate MST measurements and global fits of 5.1 ± 0.6 μM for LIC2-C+BICD2, 4.2 ± 0.5 μM for pLIC2-C+BICD2, and 43.5 ± 22.3 μM for pLIC2-C+BICD2 in the presence of Pin1 (1:100) (Supplemental Fig. 7A–C).

**Table 2:** Global *K*_D_ values fit to MST response curves from NTA-labeled LIC2-C, pLIC2-C, and pLIC2-C with Pin1 (1:100) titrated with BICD2, SPDL1, and HOOK3.

NTA-Labeled Ligand	BICD2	SPDL1	HOOK3	Pin1
LIC2-C	5.1 ± 0.6 μM	2.8 ± 0.5 μM	12.4 ± 1.4 μM	N/A
pLIC2-C	4.2 ± 0.5 μM	2.3 ± 0.4 μM	11.8 ± 2.1 μM	0.88 ± 0.17 μM
pLIC2-C+Pin1 (1:100)	43.5 ± 22.3 μM	5.5 ± 2.0 μM	12.6 ± 3.1 μM	N/A

**Fig. 6:**
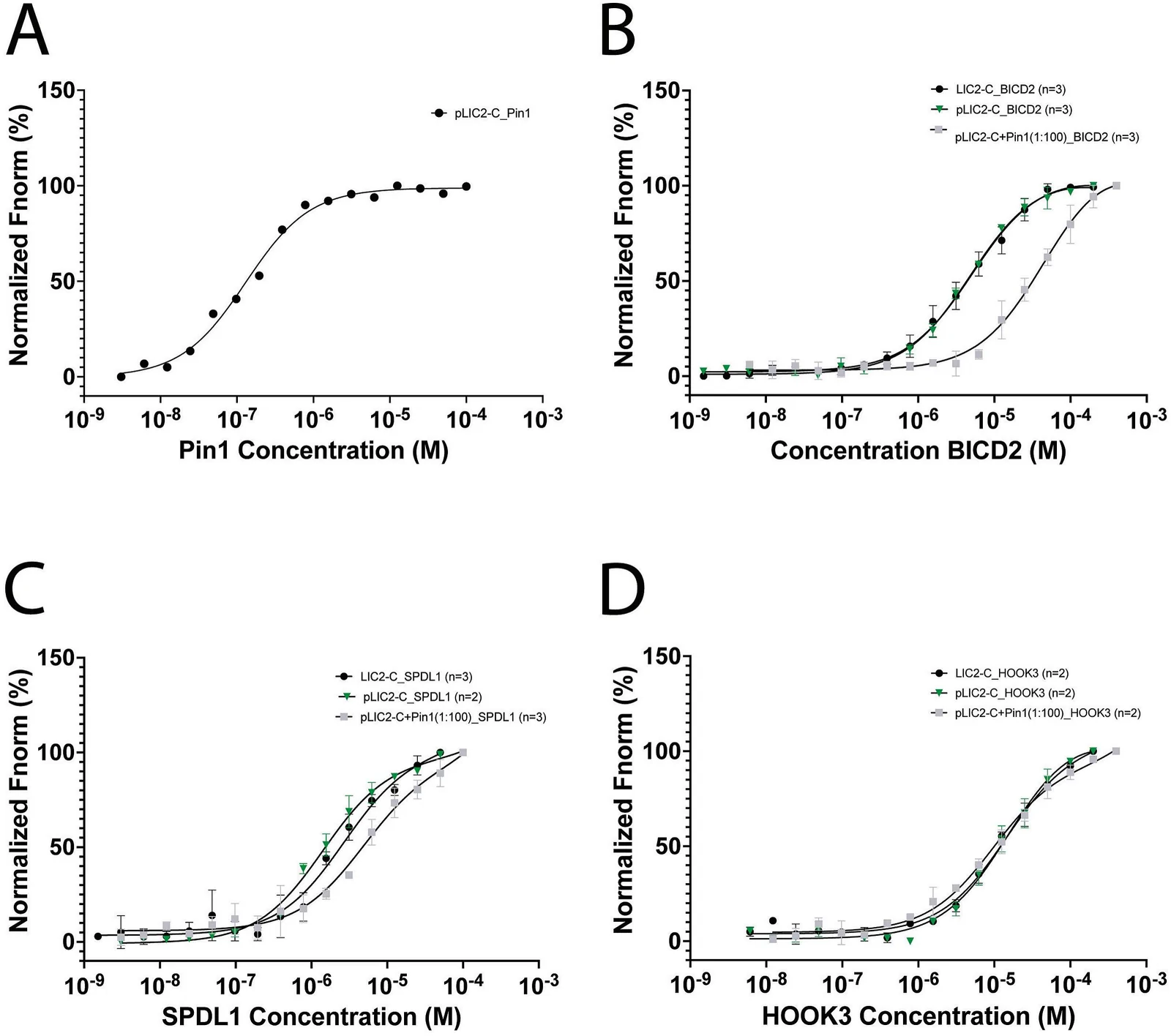
MST normalized response curves of NTA-labeled LIC2-C/pLIC2-C with BICD2, SPDL1, HOOK3, and Pin1. A) MST response curve of 50 nM pLIC2-C titrated with Pin1 (no 6xHis). B) A series of MST response curves normalized and globally fit to be plotted on the same scale of LIC2-C titrated with BICD2 (black circles), pLIC2-C titrated with BICD2 (green triangle), pLIC2-C with Pin1 (1:100), and titrated with BICD2 (grey squares). C) A series of MST response curves normalized to the same scale and globally fit: LIC2-C titrated with SPDL1 (black circle), pLIC2-C titrated with SPDL1 (green triangle), and pLIC2-C with Pin1 (1:100) and titrated with SPDL1 (grey square). D) A series of MST response curves normalized and globally fit to be plotted on the same scale of LIC2-C titrated with HOOK3 (black circle), pLIC2-C titrated with HOOK3 (green triangle), and pLIC2-C with Pin1 (1:100) and titrated with HOOK3 (grey square). All curves were fitted with a one-site Langmuir binding isotherm. (For interpretation of the references to colour in this figure legend, the reader is referred to the web version of this article.)

Using replicate MST measurements and global fits for SPDL1 we calculated *K*_D_ values of 2.8 ± 0.5 μM for LIC2-C+SPDL1, 2.3 ± 0.4 μM for pLIC2-C+SPDL1, and 5.5 ± 2.0 μM for pLIC2-C+SPDL1 in the presence of Pin1 (1:100) (Supplemental Fig. 7D–F). Lastly, we performed replicate MST measurements and global fits with HOOK3 to calculate *K*_D_ values of 12.4 ± 1.4 μM for LIC2-C+HOOK3, 11.8 ± 2.1 μM for pLIC2-C+HOOK3, and 12.6 ± 3.1 μM for pLIC2-C+HOOK3 in the presence of Pin1 (1:100) (Supplemental Fig. 7G–I). When comparing the *K*_D_s obtained by MST for LIC2-C with those previously determined by SPR and ITC for LIC1-C, we find strong agreement (Celestino et al., 2019; Lee et al., 2018a).

Interestingly, these data indicate that the *K*_D_ of each adaptor did not significantly vary between titrations with LIC2-C and pLIC2-C. This was surprising because the addition of phosphates across LIC2-C could influence charge-mediated interactions between the adaptors and LIC2. Given the relatively tight *K*_D_ of Pin1 to pLIC2-C, we used a 1:100 ratio of pLIC2-C+Pin1 in our MST binding experiments to ensure full saturation of pLIC2-C with Pin1 and subsequently to measure the *K*_D_ between the adaptors and pLIC2-C. The *K*_D_ value of BICD2 to pLIC2-C in the presence of Pin1 amounts to an order of magnitude difference compared to when Pin1 is absent. For SPDL1 and HOOK3 however, we observed no significant difference in the *K*_D_ to LIC2-C, pLIC2-C, or pLIC2-C in the presence of Pin1.

## 3 Discussion

Cytoplasmic dynein has two LIC isoforms, LIC1 and LIC2. Both isoforms have disordered C-termini, and LIC2-C remains the least characterized isoform. Here, using NMR and mass spectrometry, we identified five main Cdk1/cyclin B phosphorylation sites in LIC2-C: residues 376, 383, 391, 407, and 446. We also identified an additional 12 phosphorylation sites by mass spectrometry that were not detected by NMR, suggesting they were low-abundance and could only be detected by mass spectrometry due to its high sensitivity.

A sequence alignment of LIC1-C_388–523_ and LIC2-C_374–497_ indicates 65.7% sequence homology, and nearly complete homology at the Helix-1 and Helix-2 regions of transient α-helicity. Based on previous work that identified phosphorylation sites S398, S405, and T408 in LIC1-C and showed through mutants that these sites were relevant for cellular functions and Pin1 recruitment, it is not surprising that some of these sites were conserved in LIC2-C (Kumari et al., 2021b). LIC1-C site S398 does not directly correspond with a LIC2-C site, but LIC1-C sites S405 and T408 do align with LIC2-C sites S391 and T394. We identified S391 as a phosphorylated site (pS391) by orthogonal techniques and showed by NMR that its peak decreases in intensity upon Pin1 addition, suggestive of binding. We also identified T376 as a phosphorylation site (pT376) by NMR, which aligns with the LIC1-C site T389, however it is not followed by a proline, which would prevent T389 in LIC1-C from being efficiently phosphorylated by CDK1/Cyclin B. According to our NMR data, the pT376 peak in pLIC2-C entirely disappears upon Pin1 addition, suggesting that it is being bound by Pin1 and may be important for Pin1 recruitment to LIC2-C, but not LIC1-C. Several other identified phosphorylated residues in LIC2-C overlapped with potential phosphosites in LIC1-C, but their roles and degrees of phosphorylation are unclear.

Pin1 is unique among human PPIases in possessing two structurally distinct domains that can independently recognize the canonical pSer/pThr–Pro motif (K Ping et al., 1996; Ranganathan et al., 1997; Yaffe et al., 1997; Greenwood et al., 2011). However, only the PPIase domain has isomerization activity, while the WW domain typically has a higher affinity but is not enzymatically active (Born et al., 2019). We showed that pLIC2-C, but not LIC2-C, interacts with the peptidyl-prolyl cis-trans isomerase Pin1, although it is currently unclear which Pin1 domain is employed.

We found that phosphorylation of LIC2-C results in two clearly defined major and minor states in the Helix-1 region, as evidenced by peak splitting for several residues, including E426–S431, L437, and S438. Binding of Pin1 to pLIC2-C increases the intensity of the minor-state peaks, indicating a shift in the equilibrium between these two populations. This effect was entirely dependent on the phosphorylation state of pLIC2-C; a lower phosphorylation efficiency led to poorer Pin1 recruitment and, subsequently, a lower minor state-population. Given that Pin1 is a peptidyl-prolyl cis/trans isomerase that preferentially recognizes phosphorylated SP/TP motifs, these two states may reflect distinct proline trans and cis isomers, with Pin1 altering their relative populations. *Cis*-Adopting prolines, which can be drastically affected by phosphorylation and specific protein-protein interactions, play an important role in local structural dynamics and in mediating long-range contacts in IDPs (Mateos et al., 2020). However, further experiments are needed to test this hypothesis.

Previous work with LIC1-C and other constructs has shown that adaptors interact with the transient Helix-1, with some contacts N-terminal to Helix-1 and weaker contacts in Helix-2 (Celestino et al., 2019). One such adaptor, HOOK3, binds the Helix-1 of LIC1 after undergoing a conformational change to produce a conserved hydrophobic cleft, and LIC1_433–458_ ITC titrations into adaptors BICD2_1–98_ and Spindly_1–142_ (SPDL1) coiled-coil dimers were shown to fit best to a two binding-site isotherm (Lee et al., 2018a). Despite this, and the fact that BICD2 and SPDL1 share a similar CC1-Box to engage LIC1, the titration experiments performed indicated that the first site of SPDL1 has a mild endothermic character. In contrast, the second shows a strong exothermic character, unlike BICD2, where both sites exhibited exothermic character, suggesting potentially different conformations or binding sites between the adaptors (Lee et al., 2018a).

We showed that LIC2-C interacts with the N-terminus of the adaptor BICD2 predominantly via Helix-1 but also Helix-2. We also showed that SPDL1 engages Helix-1 and forms weaker contacts with Helix-2. Uniquely, HOOK3 was found to engage only Helix-1 and did not significantly interact with Helix-2. Phosphorylation did not interfere with LIC2-C secondary structure propensity, but it did result in increased intramolecular contacts in the Helices and the segment connecting them, suggesting an overall compaction of LIC2-C.

Phosphorylation of LIC2-C influenced the degree to which BICD2 contacted Helix-2 but not Helix-1 and did not influence SPDL1 or HOOK3 interactions with pLIC2-C. BICD2 and HOOK3 binding to pLIC2 in the presence of Pin1 appeared to favor the major state, seen as peak disappearances, while the minor state peaks remained. SPDL1, however, did not clearly exhibit this behavior. Neither NMR HSQC titration experiments with SPDL1 and LIC2 nor pLIC2 revealed peak splitting in residues E426-S431, L437, or S438; however, when SPDL1 was added to pLIC2-C with Pin1, we observed the appearance of the minor state peaks, but neither the minor nor major state peaks completely disappeared.

Our MST titration experiments corroborated some of these findings, most notably where BICD2 experienced an order of magnitude weaker *K*_D_ in the presence of Pin1 (from ∼5 down to ∼45 μM), while SPDL1 and HOOK3 were not significantly affected. This observation for BICD2 is consistent with the NMR results, which showed that Pin1 shifts the equilibrium toward the minor state that does not bind BICD2, thereby reducing the overall binding affinity.

In the case of HOOK3, we observed peak splitting during titrations with LIC2-C, pLIC2-C, and pLIC2-C+Pin1. This suggests that HOOK3 may also preferentially bind the major state, leaving the minor state unbound and revealing preexisting structural heterogeneity that is stabilized upon HOOK3 binding. Furthermore, since we did not observe a significant change in *K*_D_ for HOOK3 to LIC2-C, pLIC2-C, and pLIC2-C+Pin1 (all approximately 12 μM), a sufficient population of the pLIC2 major state-conformer may persist in the presence of Pin1 which could explain indistinguishable changes in thermophoretic responses for a weaker binder such as HOOK3. Although our NMR results suggest BICD2 and HOOK3 prefer binding to the major state of pLIC2, they experience different impacts to their affinities upon addition of Pin1 as measured by MST. This may be explained by the fact that HOOK3 has a different binding domain (a HOOK domain instead of a CC1 box), potentially resulting in a different binding mode.

Uniquely, NMR titrations of SPDL1 into LIC2-C and pLIC2-C resulted in no minor state-conformer peaks, while still exhibiting a decrease in major state-conformer peaks. SPDL1 titrations into pLIC2-C+Pin1 produced peaks of both minor and major state, though neither completely disappeared. In combination with our MST results indicating no significant change in *K*_D_ across experimental conditions (2.5–5.5 μM), we propose that SPDL1 may be more conformationally tolerant than the other adaptors and is capable of binding both the major and minor LIC2/pLIC2 states to some extent. These results shed light on a potential effect of Pin1 in modulating LIC2 function, where it could act as a negative regulator during mitosis and in the presence of phosphorylation, to reduce the total population of LIC2 available for interaction with BICD2 in favor of engagement with other adaptors (Fig. 7). Of note, Pin1 does not directly compete with BICD2, as SPDL1's and HOOK3's binding is not influenced by Pin1 and all these adaptors share the same binding site on LIC2.

**Fig. 7:**
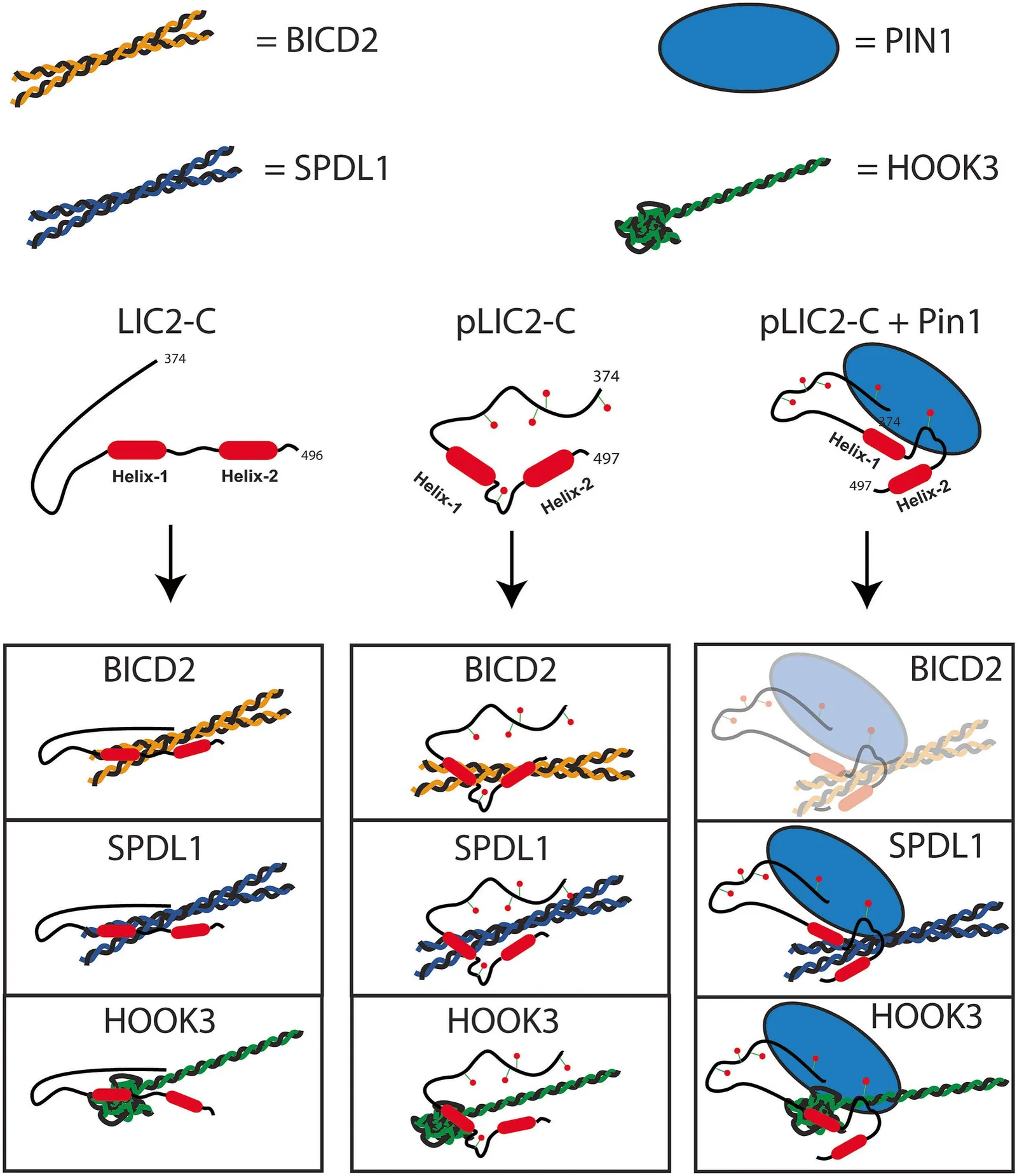
Pin1 negatively regulates BICD2 binding to pLIC2-C indirectly promoting interactions with other adaptors such as SPDL1 and HOOK3. The faded LIC2-BICD2 complex indicates reduces affinity. The proximities of Helices 1 and 2 to the adaptors reflect the interaction strength. Note that the BICD2 and SPDL1 N-termini are dimeric because of their coiled-coil region, while the N-terminal HOOK domain of HOOK3 is monomeric.

Whether the minor or major states impact LIC2 interactions with adaptors only through local geometry changes or also through long-range rearrangements remains unclear. It is possible that an initially local rearrangement repositions the Helices relative to each other. If the driving force behind the two states is indeed proline isomerization as speculated above, such a rearrangement could be caused by a proline conformational switch, in which the segments up- and downstream of proline may change their projection angle by ∼100–140 degrees. It is also possible that the two Pin1 binding sites bind to different sites on LIC2 simultaneously, thereby repositioning remote segments (Shoemaker et al., 2000; Daum et al., 2007; Innes et al., 2013), possibly through allosteric effects across the two Pin1 domains (Peng, 2015; Born et al., 2022; Born et al., 2021).

It is known that protein phosphorylation increases dramatically in the cytoplasm and throughout the cell upon entry into and during mitosis. This phosphorylation is primarily driven by the activation of mitotic kinases, such as CDK1/Cyclin B and PLK1. We propose that phosphorylation of LIC2 and potentially LIC1 by CDK1/Cyclin B occurs during the cell's transition into mitosis and enables Pin1 binding. As Pin1 binds, it shifts the conformational equilibrium of phosphorylated LIC2, possibly driven by catalysis of the isomerization of a proline or prolines such as the one located in the linker between Helix-1 and Helix-2, downregulating the recruitment of BICD2 to LIC2 and thereby upregulating the recruitment of other adaptors.

SPDL1 is required for the localization of dynein and dynactin to the mitotic kinetochore, which is built onto the centromere of condensed chromosomes during prophase/metaphase (Henen et al., 2021; Yamamoto et al., 2008). This mechanism may explain why mitotic phosphorylation of C-terminal region of LIC1 at its three Cdk1 sites is required for proper mitotic progression, dynein loading onto prometaphase kinetochores, and inactivation of the spindle assembly checkpoint (Kumari et al., 2021b).

Interestingly, a previous study showed that during mitosis phosphorylatable LIC1 showed a complete loss of BICD2 binding, while the unphosphorylatable mutant still retained binding (Kumari et al., 2021b). Furthermore, incomplete Golgi fragmentation was observed with a LIC1 phospho-deficient mutant but not the wildtype LIC1 (Kumari et al., 2021b; Neveling et al., 2013). Indeed, downregulation of BICD2 binding to LIC2 and LIC1 during mitosis would serve as a biologically relevant and critical regulatory mechanism that is phosphorylation and Pin1 dependent.

The ability for different LIC isoform bearing dynein complexes to negatively select for interactions with an adaptor at various cell cycle points may help explain the lack of cytoplasmic dynein forms, and the variability in adaptor LIC-interacting sequences. It would also be interesting to know if the phosphorylation of adaptors or other dynein subunits recruits Pin1, and if such modifications would influence interactions with different LIC states. Structural characterization of the dynein machinery and cargo adaptors expands beyond the scope of understanding normal cellular function to pathogens like adenovirus which directly interact with dynein through its intermediate chain (IC) and light intermediate chain (LIC) (Bremner et al., 2009). It is possible that structural rearrangements of LIC, which are influenced by phosphorylation, could be a mechanism that allows activation and inactivation of virus transport, and could therefore serve as a potential target for intervention.

## 4 Materials and methods

### 4.1 Protein expression and purification

**Pin1:** Wild-type Pin1 was cloned into the pET28a(+) vector with a 6xHis tag and kanamycin resistance. The plasmid was transformed and expressed in the *Escherichia coli* strain BL21 (DE3). In brief, overnight cultures were resuspended in 1 L of LB growth media and grown at 37 °C while shaking at 220 rpm until the optical density at 600 nm reached 0.8, and then induced with 1 mM isopropyl-β-D-thiogalactoside. Induced cells were then grown overnight at 20 °C, shaking at 220 rpm. Cells were harvested by centrifugation at 4 °C for 20 min at 4000*g*. For protein purification, cells were resuspended in 50 mM potassium phosphate with 1 mM dithiothreitol, 0.3 mM phenylmethylsulfonyl fluoride, and 25 mM imidazole at pH 7.5. Cells were lysed by sonication (4 × 30 s) at 4 °C and then centrifuged for 45 min at 52,000*g* at 4 °C. Cell lysate was filtered and loaded onto a pre-equilibrated nickel nitrilotriacetic acid column (GE Healthcare) on an AKTA Pure at 4 °C. The His-tagged protein was first washed with low imidazole buffer (20 mL) and eluted with 30 mL of high imidazole buffer. Pin1 was concentrated using a 3000 NMWL cutoff, then purified using a Superdex 75 10/300 GL size-exclusion column in 50 mM sodium phosphate and 150 mM sodium chloride buffer at pH 6.5.

Pin1 Thrombin Cleavage: 51 U of Thrombin (Crude, Bovine, 2kU, catalog number BP25432) was incubated with 39.285 μg of Pin1(1–183)::6xHis in 1 mL of 1× PBS (137 mM NaCl, 2.7 mM KCl, 10 mM NaH2PO4, 1.8 mM NaHPO4, pH 8.0). Cleavage reaction was held constant at 30 °C and gently agitated overnight (∼18 h). Reaction was diluted to a final volume of 5 mL in low-imidazole buffer (20 mM HEPES, 200 mM NaCl, 1 mM EGTA, 1 mM MgCl2, 1 mM NaN_3_, 20 mM imidazole, pH 7.3) and purified using an AKTA FPLC and HisTrap FF column. Samples were eluted using a High-imidazole buffer (20 mM HEPES, 20 mM NaCl, 1 mM EGTA, 1 mM MgCl2, 1 mM NaN_3_, 200 mM imidazole, pH 7.3). Purified protein was buffer exchanged into NMR buffer using an Amicon Ultra-15 3 kDa MWCO Centrifugal Filter (Millipore Sigma, Catalog# UFC9003) and frozen at −80 °C until needed.

**LIC2-C:** LIC2-C WT or G396C constructs bacterial expression vectors were transformed into the *Escherichia coli* strain BL21. Expression was induced with 0.1 mM IPTG at 20 °C overnight at an OD600 of 0.9, and cells were harvested by centrifugation for 20 min at 4000*g*. Clarified GST::LIC2-C::6xHis cell lysate was filtered and loaded onto a pre-equilibrated nickel nitrilotriacetic acid column (GE Healthcare) on an AKTA Pure at 4 °C. The His-tagged protein was first washed with low imidazole buffer (20 mL) and eluted with 30 mL of high imidazole buffer. GST::LIC2-C::6xHis was resuspended in a PreScission Protease Cleavage Buffer (50 mM Tris-HCl, 150 mM NaCl, 1 mM EDTA, 1 mM DTT, pH 7) and cleaved with PreScission Protease while shaking overnight at 4 °C. Cleaved glutathione was removed by repeated purification onto a pre-equilibriated nickel nitrilotriacetic acid column on an AKTA pure at 4 °C. Eluted LIC2-C::6xHis was subjected to size-exclusion chromatography using a Superdex 75 increase 10/300 GL column (GE Healthcare) in NMR buffer. Aliquots of purified LIC2-C were frozen at −80 °C.

**Adaptors:** Three adaptor constructs have been expressed in this study: BICD2 (residues 2–422), SPDL1 (residues 2–359), and HOOK3 (residues 2–239). The adaptors bacterial expression vectors were transformed into the *Escherichia coli* strain BL21. Expression was induced with 0.1 mM IPTG at 20 °C overnight at an OD_600_ of 0.9, and cells were harvested by centrifugation for 20 min at 4000*g*. For purification of cargo adaptors, bacterial pellets were resuspended in lysis buffer B (50 mM HEPES, 250 mM NaCl, 10 mM imidazole, 0.1% Tween 20, 1 mM DTT, 1 mM PMSF, 2 mM benzamidine-HCl, 1 mg/mL lysozyme [pH 8.0]), disrupted by sonication, and clarified by centrifugation at 34,000*g* for 45 min. Clarified 6xHis::MBP::adaptor supernatants were filtered and loaded onto a pre-equilibrated nickel nitrilotriacetic acid column (GE Healthcare) on an AKTA Pure at 4 °C. The loaded His-tagged adaptor was first washed with low imidazole buffer (20 mL) and eluted with 30 mL of high imidazole buffer. Fractions containing the recombinant proteins were pooled, and DTT was added to a final concentration of 5 mM before shaking overnight at 4 °C with TEV protease to cleave the 6xHis::MBP moiety. Post-TEV-treated adaptors were buffer-exchanged into low-imidazole buffer before being repurified by Ni-affinity chromatography. Bound 6xHis::MBP fractions were retained on the Ni-Affinity resin. At the same time, adaptors were collected as flowthrough and pooled before being subjected to size-exclusion chromatography using a Superdex 75 increase 10/300 GL column (GE Healthcare) in NMR buffer. Aliquots of purified adaptors were frozen at −80 °C.

### 4.2 *In vitro* phosphorylation

160 μg isotopically labeled (^15^N or ^15^N/^13^C labeled) bacterially purified LIC2-C were incubated with 8 μg of recombinant full length human Cdk1/cyclin B (Eurofins, 14–450 or Thermo Fisher Scientific, PV3292) with 800 μM ATP (catalog number 50994923) and 1× protein kinase buffer (50 mM Tris-HCl, 10 mM MgCl_2_, 0.1 mM EDTA, 2 mM DTT, 0.01% Tween-20, pH 7.5) in a total volume of 4.8 mL. Phosphorylation control samples used for mass spectrometry analyses contained each component except Cdk1/cyclin-B. The reaction mixture was incubated in a water bath at 30 °C for 2 h followed by buffer exchange and concentration with an Amicon Ultra-15 3 kDa MWCO Centrifugal Filter (Millipore Sigma, Catalog# UFC9003) and NMR buffer (50 mM NaP, 150 mM NaCl, 1 mM DTT, 0.02% NaN_3_, pH 6.5) to a final volume of 100–150 μL. To confirm that residual CDK1/Cyclin-B in our NMR samples was not contributing to shifts or influencing the signal, we heated 150 μL of phosphorylated LIC2-C prepared for NMR in a boiling water bath for 45 min. A control phosphorylated LIC2-C sample was also prepared for NMR but not subjected to the boiling procedure. We then performed HSQC measurements on the boiled and control samples and overlayed the spectra (Supplemental Fig. 8). This protocol has been adapted for the preparation of all the phosphorylated samples used for assignment, Pin1 titration, adaptor titration and affinity measurements. Each prepared sample has been validated using the NMR HSQC as a fingerprint.

### 4.3 PRE labeling

For MTSL spin labeling, 2.5 mL of LIC2-C G396C or pLIC2-C G396C at a concentration of 200 μM in NMR buffer was supplemented with 1 mM dithiothreitol (DTT) and incubated for 15 min at room temperature to reduce free cysteine thiols. A PD-10 desalting column was equilibrated with 25 mL of reaction buffer (100 mM sodium phosphate [NaPi], pH 8.0, 1 mM EDTA). The protein sample (2.5 mL) was loaded onto the equilibrated column, the flow-through was discarded, and the protein was eluted with 3.5 mL of reaction buffer. MTSL was then added from a 50 mM stock solution in DMSO to a fivefold molar excess relative to the protein, and the labeling reaction was incubated for 3 h at 37 °C. The reaction was quenched by addition of a twofold molar excess of β-mercaptoethanol, and any precipitated material was removed by centrifugation. The labeled protein was subsequently dialyzed against NMR buffer and concentrated to a final concentration of 140 μM for the nonphosphorylated LIC2-C and 50 μM for pLIC2-C in a volume of 140 μL for NMR measurements. For preparation of the diamagnetic reference sample, the MTSL-labeled protein was treated with DTT at a five- to tenfold molar excess relative to the protein and incubated for 15 min to reduce the MTSL nitroxide and quench the paramagnetic relaxation enhancement (PRE) effect.

### 4.4 NMR spectroscopy

^13^C- and ^15^N-labeled NMR samples were prepared in the NMR buffer and measured in a 3 mm tube. NMR spectra were acquired on BRUKER Avance NEO 600 MHz triple-resonance cryoprobe spectrometers at 25 °C. The 2D ^1^H–^15^N HSQC and 3D HNCACB and (H)N(*CA*)NNH experiments were acquired using a nonuniform sampling (NUS) scheme generated by the NUS@HMS scheme generator (Hyberts et al., 2012). In the 2D ^1^H–^15^N HSQC, the number of points used was 1024 complex data points in the direct dimension and 50% sampling of the original 200 complex points in the indirect ^15^N dimension. The spectral widths were 13.7 and 35 ppm for the ^1^H and ^15^N dimensions, respectively, with a recycle delay of 1.3 s and 16 scans. All HSQC spectra were assigned using previously assigned 3D spectra (Henen et al., 2023).

In the HNCACB experiment, 1024 complex data points in the direct dimension were used, and 30% sampling of the original 256 and 92 complex points in the indirect ^15^N and ^13^C dimensions, respectively (Hyberts et al., 2012). The spectral widths were 13.7, 35, and 80 ppm for the ^1^H, ^15^N, and ^13^C dimensions, respectively, with a recycle delay of 1s and 16 scans.

In the (H)N(*CA*)NNH experiment, 1024 complex data points in the direct dimension were used, and 30% sampling of the original 100, and 256 complex points in the two indirect 15 N dimensions (Hyberts et al., 2012). The spectral widths were 13.7 and 35 for the ^1^H and ^15^N dimensions, respectively, with a recycle delay of 1 s and 32 scans.

The 3D experiment (H)CC(*CO*)NH was measured using uniform sampling and 1024, 20, and 49 complex points in the ^1^H, ^15^N, and ^13^C dimensions, respectively. The spectral widths were 16, 35, and 80 ppm in the ^1^H, ^15^N, and ^13^C dimensions, respectively, with a recycle delay of 1 s and 16 scans. A DIPSI spinlock of 12 ms duration was used to transfer magnetization between the side-chain carbons.

Further data processing and visualization were conducted using NMRpipe/NMRDraw (Delaglio et al., 1995). The backbone resonance assignment was carried out using the CCPNmr Analysis software v2.5.1 (Vranken et al., 2005). The assignment of LIC2-C has previously been deposited in the BMRB database under the BMRB accession code 51890 (total 93.3%; 99 ^13^Cα, 87 ^13^Cβ, and 99 ^1^H/^15^N resonances) (Henen et al., 2023). The assignment of pLIC2-C has been achieved by shifting the resonances of LIC2-C in the spectra of the experiments above and deposited under the accession code 54003.

The chemical shift perturbations were calculated using the following equation:

$$\mathrm{CSP}=\sqrt{\left(0.15*∆N^{2}\right)+\left(∆H^{2}\right)}$$

For comparison of peak intensities between LIC2-C and pLIC2-C or between PRE-labeled and reduced LIC2-C/pLIC2-C, samples were prepared at identical protein concentrations in NMR buffer, and spectra were acquired using identical NMR acquisition parameters. Peak intensities were extracted from each spectrum using CCPNmr Analysis (Vranken et al., 2005). For each assigned residue, the peak intensity ratio between pLIC2-C and LIC2-C was calculated to assess residue-specific changes in signal intensity.

### 4.5 Mass spectrometry

20 μg of Control sample (-CDK1/Cyclin B) and 20 μg of Experimental sample (+CDK1/Cyclin B) were in-solution digested for LC-MS analysis. Briefly, individual protein samples were reduced by adding 120 mM TCEP in H_2_O to a final concentration of 5 mM and incubating at room temperature for 20 min. Glu-C enzyme, sequencing grade (Promega, Madison, WI), was added to the sample at a 1:25 enzyme-to-substrate ratio. Samples were incubated at 37 °C overnight (∼16 h) to complete the digestion. Peptides from the digestion were desalted using Pierce C18 Spin Tips (ThermoFisher, Waltham, MA) according to the manufacturer's instructions. LC-MS/MS analysis of the desalted peptide samples were analyzed on a nano-UHPLC (nanoElute, Bruker Daltronics) coupled to a timsTOF SCP mass spectrometer (Bruker Daltonics). A PepSep 25 cm × 150 cm, C18 1.5 μm (Bruker Daltonics) column heated to 50 °C was used for the separation. Peptides were separated on a stepped, 90 min gradient utilizing 0.1% (*v*/v) formic acid (mobile phase A) and acetonitrile (mobile phase B) at a flow rate of 750 nL/min. The gradient steps consisted of sequential steps consisting of: (a) 4 to 25% B in 60 min, (b) 25 to 35% B in 20 min, and (c) 35 to 95% B in 10 min.

The mass spectrometer was operated in PASEF mode with an accumulation time of 75 ms and 5 PASEF MS/MS scans per topN acquisition cycle. MS and MS/MS spectra were recorded for a *m/z* range of 100 to 1700, and ion mobility was scanned from 0.6 to 1.4 Vs/cm^2^. Precursors for data-dependent acquisition were isolated within ±1 and fragmented with an ion mobility-dependent collision energy, which was linearly increased from 20 to 65 eV in positive mode. Low-abundance precursor ions with an intensity above a threshold of 1000 counts but below a target value of 12,500 counts were repeatedly scheduled and otherwise dynamically excluded for 0.2 min. Downstream identification, filtering, and validation of LC-MS data were managed using Fragpipe version 23.0. MSFragger version 4.4.0 was used for the database search of the LC-MS data versus a fasta format file containing the amino acid sequence of the LIC2-C construct, as well as decoy and common contaminant proteins. Search settings were as follows: GluC, enzymatic cleavage at DE, 2 missed cleavages allowed, 20 ppm MS1 and MS2 tolerances, 3 maximum variable mods, phosphorylation at STY (+79.96633), N-terminal acylation (+42.0106), and M oxidation (+15.9949). LIC2-C was ^15^N labeled for NMR analysis, so the following fixed modifications were included as well: R (+3.98814), H (+2.91105), KQN (+1.99407), and all other amino acids (+0.997035). PTMProphet was used for PTM localization, and the FDR filter was set to 0.01. Label-free quantification was accomplished using IonQuant version 1.11.20.

### 4.6 MST binding measurements

Binding measurements were carried out on a NanoTemper Monolith NT.115 p instrument (NanoTemper Technologies) at 25 °C using medium power and 10% excitation power (auto-detect-pico-red). LIC2-C(374–497)::6xHis or pLIC2-C(374–497)::6xHis was fluorescently labeled by interacting 100 μL protein solution (200 nM concentration) with 100 μL Red-Tris NTA dye (100 nM concentration). For pLIC2-C and Pin1 MST experiments, thrombin-cleaved Pin1 was added to the NTA labeling reaction to final concentrations of 1 μM for the singular BICD2 measurement and 10 μM for all other adaptor measurements. 6xHis-tag removal of Pin1 was validated by MST titration experiments, in which Pin1 with a 6xHis tag was titrated into 50 nM of NTA-Dye (Supplemental Fig. 5K) and compared to a titration of Pin1 without a 6xHis tag into 50 nM NTA-Dye (Supplemental Fig. 5J). We observed a *K*_D_ of 25 nM for Pin1 with His-tag, and 200 μM for Pin1 without His-tag as a control, and therefore Pin1 without His-tag was used for the remainder of the MST titrations, while the wild-type Pin1 with an intact 6xHis-tag was used for the NMR binding experiments. The NTA-labeling reaction mixture was incubated at room temperature for 30 min on a shaker at 1500 rpm, followed by centrifugation for 10 min at 4 °C with 15,000 g. Then, 50 nM of labeled LIC2(374–497)::6xHis, pLIC2-C(374–497)::6xHis, and pLIC2-C(374–497)::6xHis+Pin1 (1 or 10 μM) and 16 two-fold dilution series of the adaptors BICD2, SPDL1, and HOOK3 were loaded into 16 premium capillaries (NanoTemper Technologies).

50 nM of labeled pLIC2-C(374–497)::6xHis was measured against 16 two-fold dilution series of thrombin-cleaved Pin1 in 16 premium capillaries (NanoTemper Technologies) to a highest concentration of 100 μM (SPDL1[2–359]:: Strep-tag II highest concentration 100 μM; BICD2[2–422]:: Strep-tag II highest concentration 200 μM; HOOK3[2–239]::Strep-tag II highest concentration 200 μM). We observed sigmoidal behavior of the fluorescence level over time, which allowed characterization of the interactions. Raw data were analyzed using the NanoTemper software (MO affinity analysis v 2.2.7) and PALMIST (version 1.5.1) for global fitting (Scheuermann et al., 2016).

## CRediT authorship contribution statement

**Michael Warchol:** Writing – original draft, Methodology, Investigation, Formal analysis, Data curation. **Charles W. Kroft:** Writing – review & editing, Formal analysis. **Jeffrey B. Krall:** Writing – review & editing, Formal analysis. **Shaun Bevers:** Writing – review & editing, Resources, Investigation, Formal analysis. **Robb Welty:** Writing – review & editing, Investigation, Formal analysis. **Morkos A. Henen:** Writing – review & editing, Supervision, Methodology, Investigation, Formal analysis, Conceptualization. **Beat Vögeli:** Writing – review & editing, Supervision, Funding acquisition, Conceptualization.

## Funding sources

This project was supported by NIH grant R35GM156171 to B.V., 10.13039/100013678University of Colorado Cancer Center Support Grant P30 CA046934, and Biomedical Research Support Shared Grant S10 OD025020.

## Declaration of competing interest

The authors declare that they have no known competing financial interests or personal relationships that could have appeared to influence the work reported in this paper.

## Data Availability

Data will be made available on request.

## Appendix A Supplementary data

Supplementary material : Details of resonance assignments of HSQC spectra of (p)LIC2-C, LC-MS analysis, G396C mutant and PRE, LIC2-C, NMR and MST titrations of LIC2-C, pLIC2-C, and pLIC2-C+Pin1 with adaptors, and sample purity.
