# Supplementary material for: Phosphorylation in the dynein light intermediate chain 2 isoform enables Pin1 binding and modulates cargo-adaptor interactions
Source: J Struct Biol X. 2026 Sep 22;14:100165. doi: 10.1016/j.yjsbx.2026.100165 (PMC13635249; doi:10.1016/j.yjsbx.2026.100165)
Supplement: Supplementary file 1 [file mmc1.pdf]

Supplemental Information

Phosphorylation in the Dynein Light Intermediate Chain 2 isoform enables Pin1 binding and modulates cargo-adaptor interactions

Michael Warchol, Charles W. Kroft, Jeffrey B. Krall, Shaun Bevers, Robb Welty, Morkos A. Henen*, Beat Vögeli*

Department of Biochemistry and Molecular Genetics, School of Medicine, University of Colorado Anschutz Medical Campus, Aurora, CO 80045, USA

*Correspondence: beat.vogeli@cuanschutz.edu; Tel.: +1 (303) 724-1627; morkos.henen@cuanschutz.edu; Tel.: +1 (210) 388 7673

KEYWORDS: dynein, dynein light intermediate chain, Pin1, DLIC2, motor adaptors, phosphorylation

*The following document includes supplemental information that could not be included in the main body of the paper.

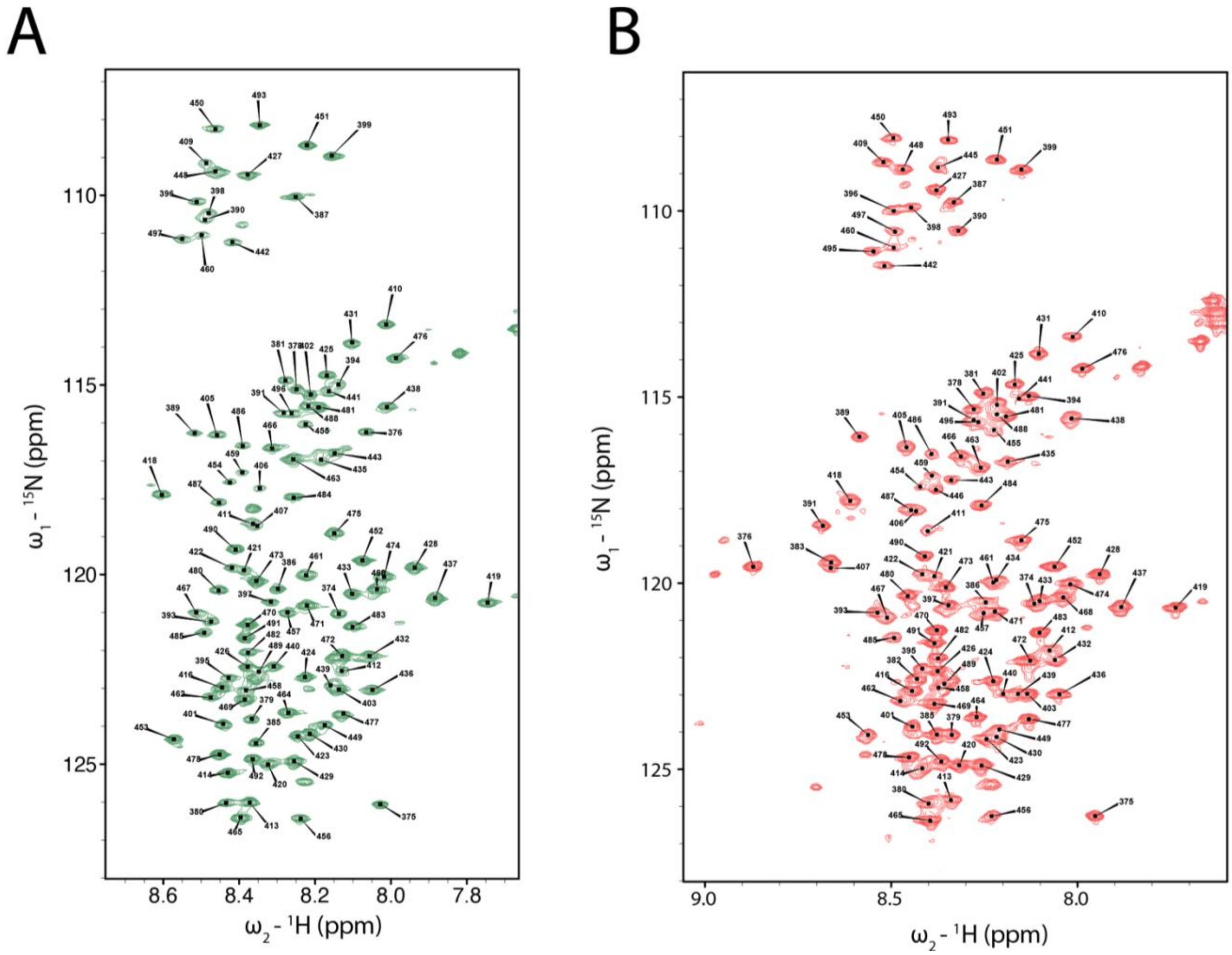

Supplemental Figure 1. Assignment strategy. A) Assignment of ^{15}N-LIC2-C performed by Henen et al. with residue numbers 374-497 excluding prolines. [1] B) Assignment of ^{15}N/^{13}C-pLIC2-C with residue numbers 374-496 excluding prolines.

A

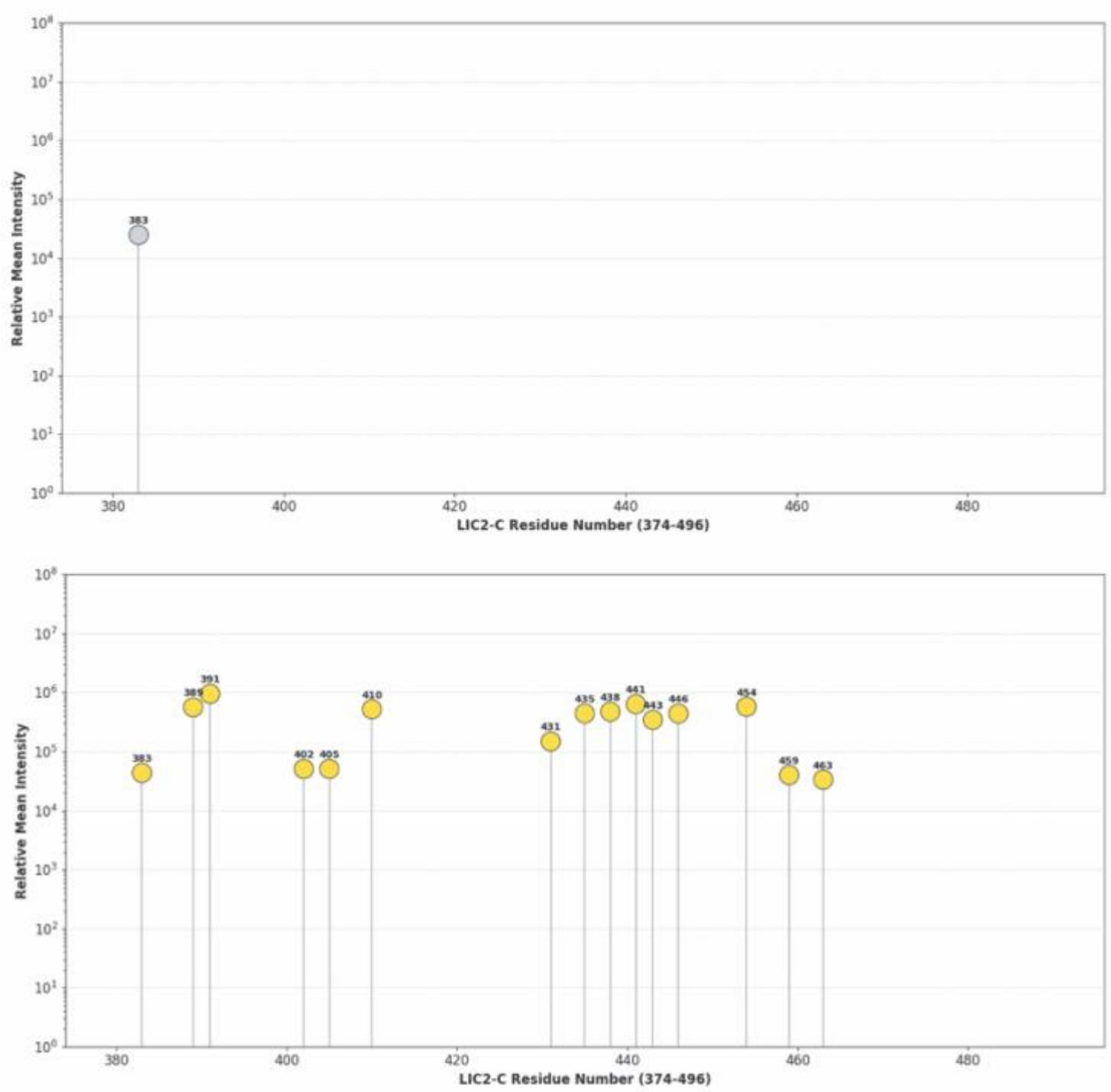

B

Position	+CDK1/Cyclin-B Intensity	-CDK1/Cyclin-B Intensity	Log2FC
S383	43457.477	24908.572	0.803
S389	559623.4	0	19.094
S391	945882.25	0	19.851
S402	50525.375	0	15.625
S405	50525.375	0	15.625
T410	519923.56	0	18.988
S431	147961.97	0	17.175
S435	439689.2	0	18.746
S438	470353.25	0	18.843
T441	633277.94	0	19.272
S443	343082.03	0	18.388
S446	437534.38	0	18.739
S454	573175.4	0	19.129
S459	39827.08	0	15.281
T463	33256.344	0	15.021

C

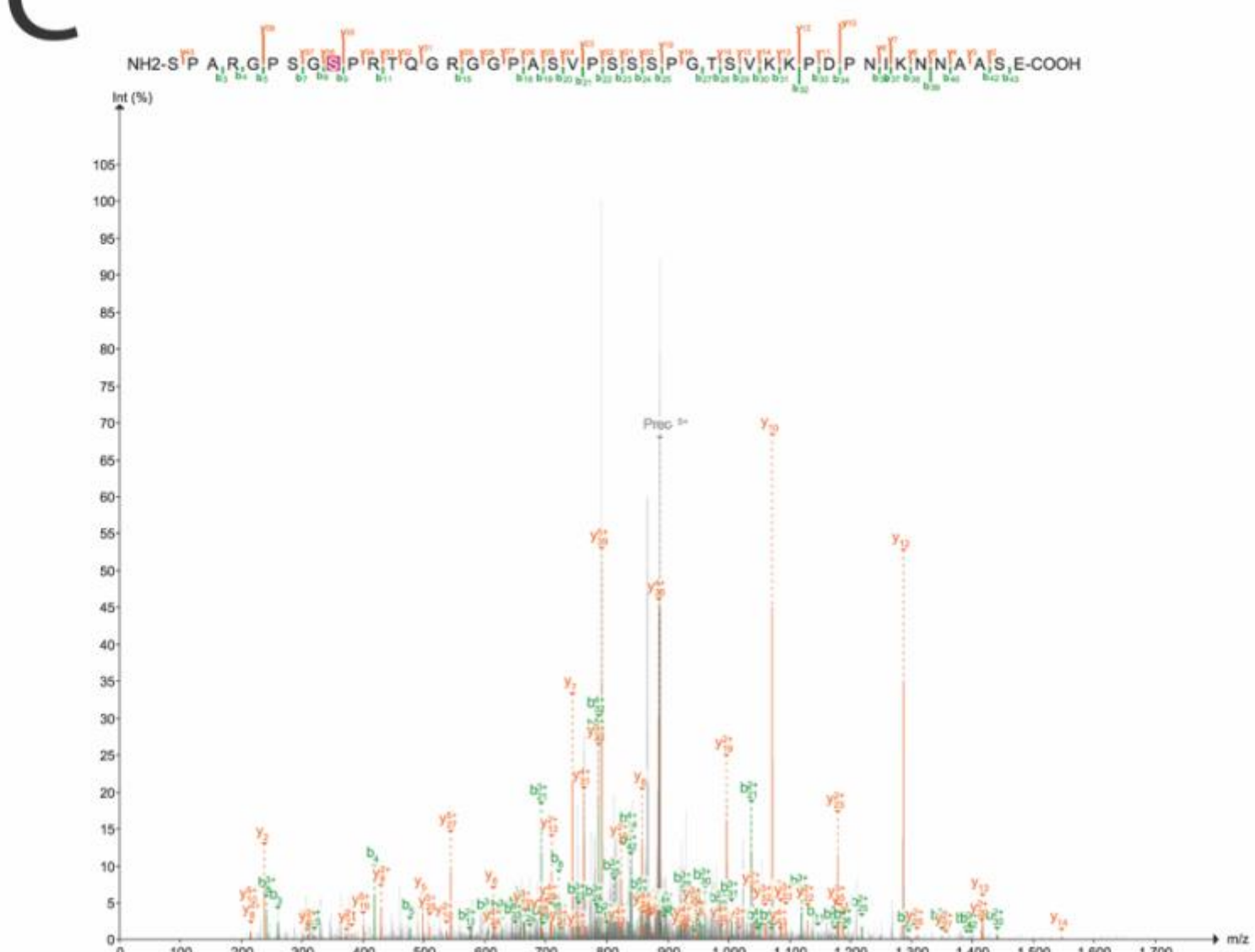

Supplemental Figure 2. Bottom-up LC-MS analysis of pLIC2-C indicates 15 phosphorylated residues. A) Control experiment of LIC2-C without CDK1/Cyclin B (top, grey) digested with GluC protease and fragment searched for phosphorylation of serines and threonines. LIC2-C sample treated with CDK1/Cyclin B (bottom, yellow) and digested with GluC protease and fragment searched for phosphorylation of serines and threonines. The mean relative abundance values of peptides containing a phosphorylated residue are plotted on a Log10 scale against the LIC2-C sequence. B) Table indicating the phosphosite position in LIC2-C, mean +CDK1/Cyclin B sample peptide intensity, -CDK1/Cyclin B sample peptide intensity, and Log2 fold change calculations. C) Fragmentation pattern of the peptide -NH2-SPARGPSGSPRTQGRGGPASVPSSSPGTSVKKPDPNIKNNAASE-COOH containing pS391.

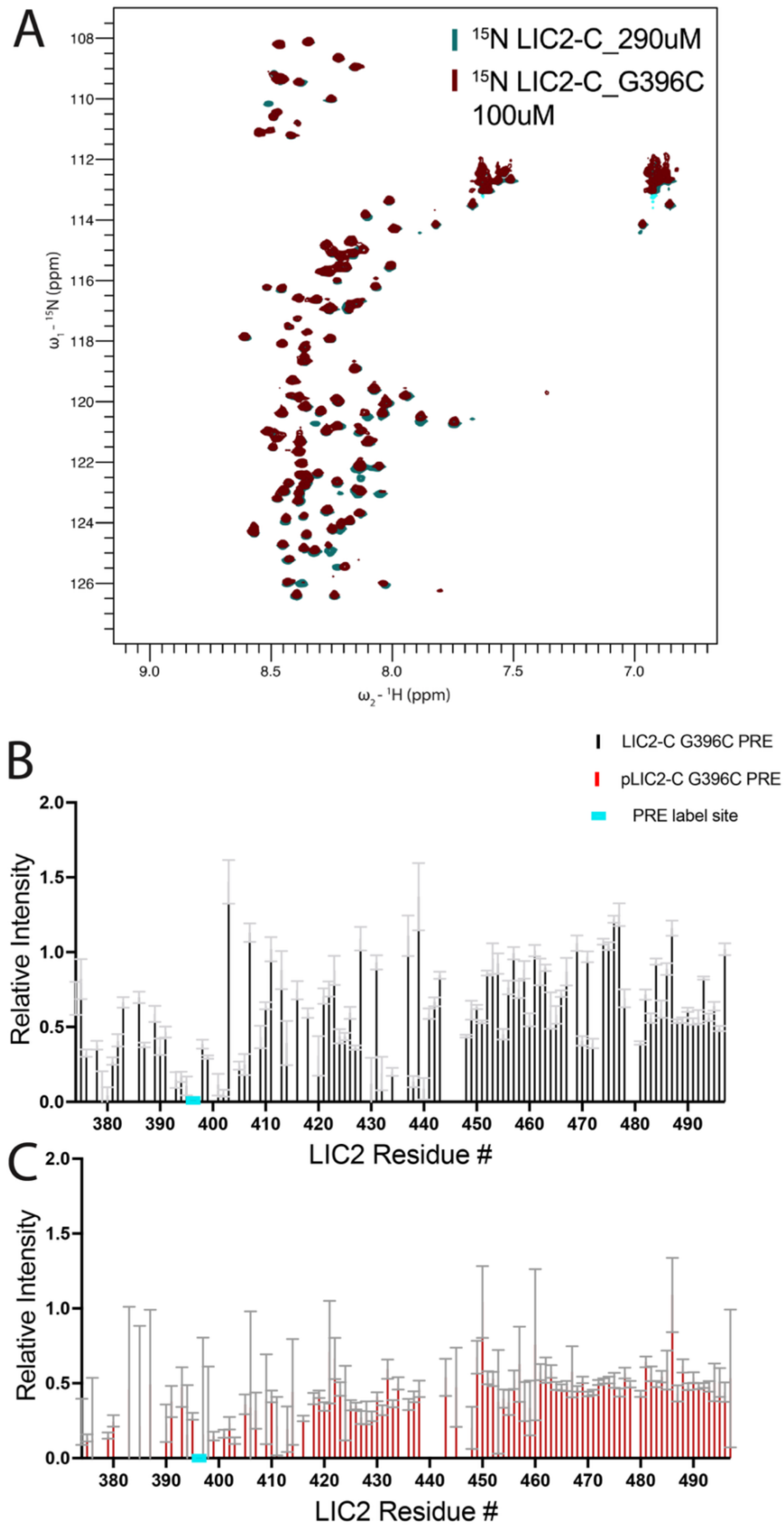

Supplemental Figure 3. Control and errors in PRE measurements. A) Structure conservation upon G396C mutation. Overlay of 290 µM ^{15}N-LIC2-C (cyan) and 100 µM ^{15}N-LIC2-C G396C mutant (brown). B) Peak height ratios of MTSL-labeled and reduced LIC2-C G396C mutants with error bars for LIC2-C and C) for pLIC2-C.

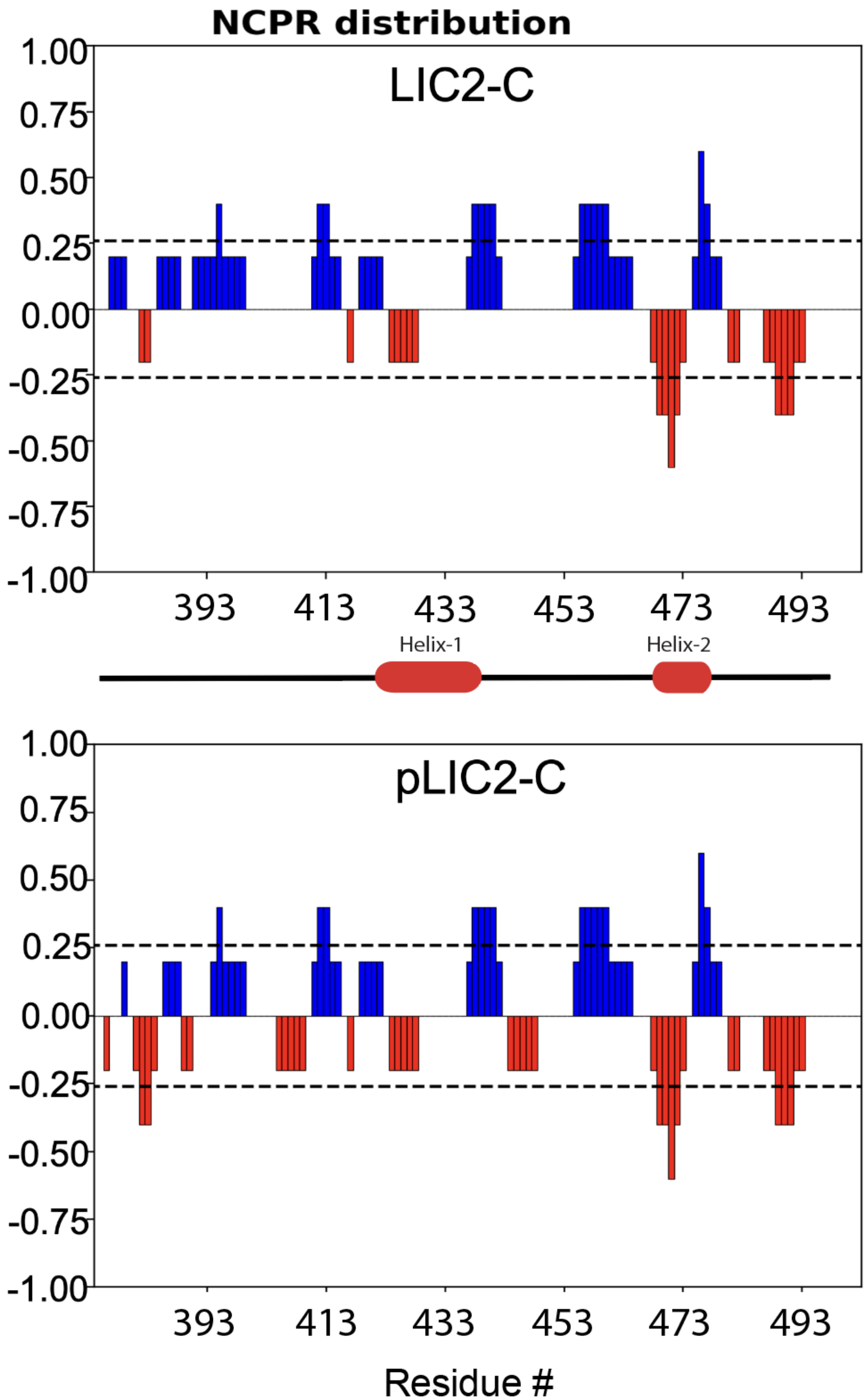

Supplemental Figure 4: Residue charge distribution of LIC2-C and pLIC2-C. Net charge distribution per residue (NCPR) for LIC2 (top) and pLIC2 (bottom), where residues 376, 383, 391, 407, and 446 were substituted with Glutamic acids. Blue and red denote positive and negatively charged residues, respectively. Figures generated with the program CIDER.[2]

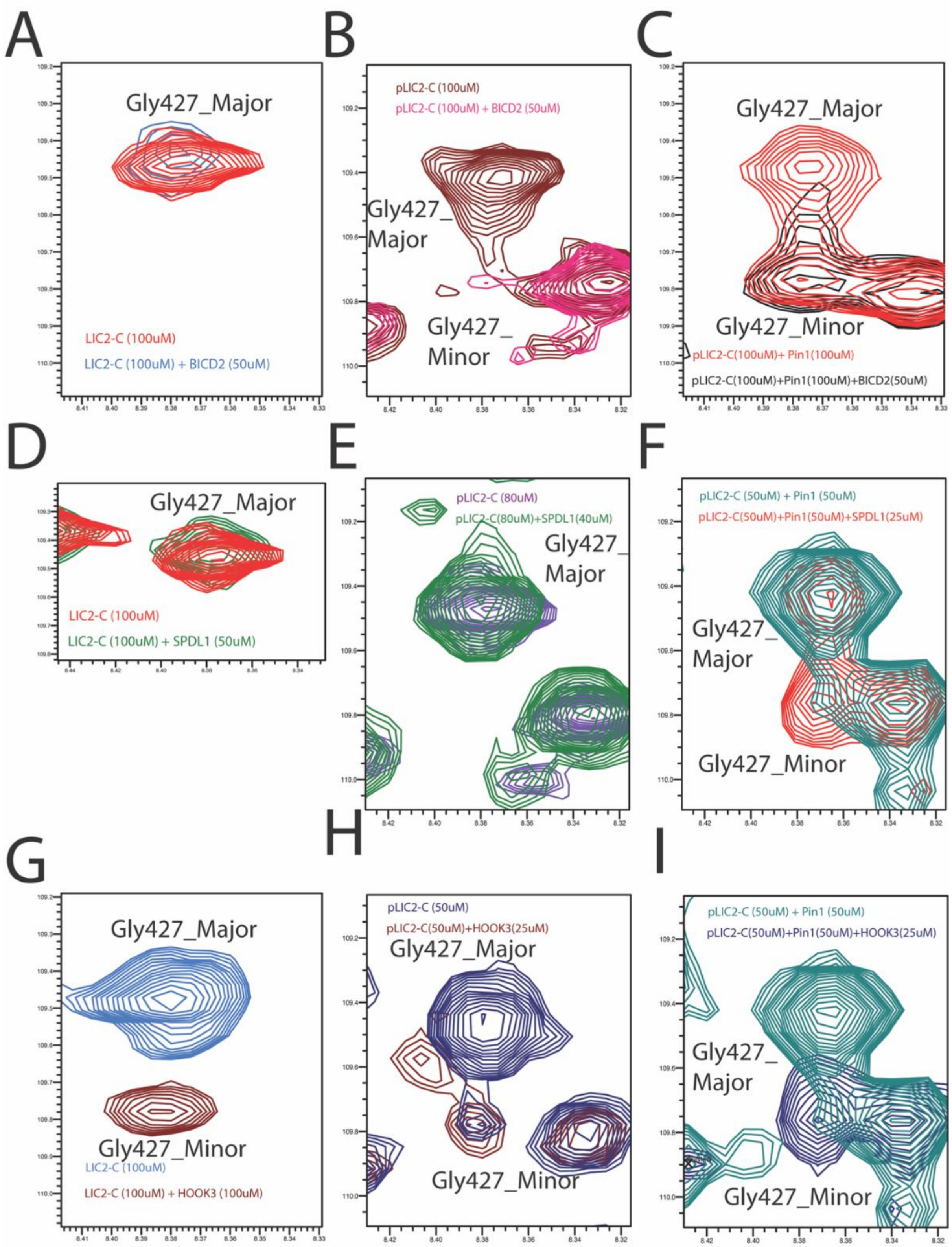

Supplemental Figure 5: BICD2 and HOOK3, but not SPDL1, preferentially engage the trans-conformer Gly427 and not the Cis-conformer Gly427. A) 100 uM LIC2-C HSQC (red) overlayed with 100 uM LIC2-C + 50 uM BICD2 HSQC (blue). B) 100 uM pLIC2-C HSQC (brown) overlayed with 100 uM pLIC2-C + 50 uM BICD2 HSQC (pink). C) 100 uM pLIC2-C + 100 uM Pin1 HSQC (red) overlayed with 100 uM pLIC2-C + 100 uM Pin1 and 50 uM BICD2 HSQC (black). D) 100 uM LIC2-C HSQC (red) overlayed with 100 uM LIC2-C + 50 uM SPDL1 HSQC (green). E) 80 uM pLIC2-C HSQC (purple) overlayed with 80 uM pLIC2-C + 40 uM SPDL1 HSQC (green). F) 50 uM pLIC2-C + 50 uM Pin1 HSQC (cyan) overlayed with 50 uM pLIC2-C + 50 uM Pin1 and 25 uM SPDL1 HSQC (red). G) 100 uM LIC2-C HSQC (blue) overlayed with 100 uM LIC2-C + 100 uM HOOK3 HSQC (brown). H) 50 uM pLIC2-C HSQC (dark blue) overlayed with 50 uM pLIC2-C + 40 uM HOOK3 HSQC (brown). I) 50 uM pLIC2-C + 50 uM Pin1 HSQC (teal) overlayed with 50 uM pLIC2-C + 50 uM Pin1 and 25 uM SPDL1 HSQC (dark blue).

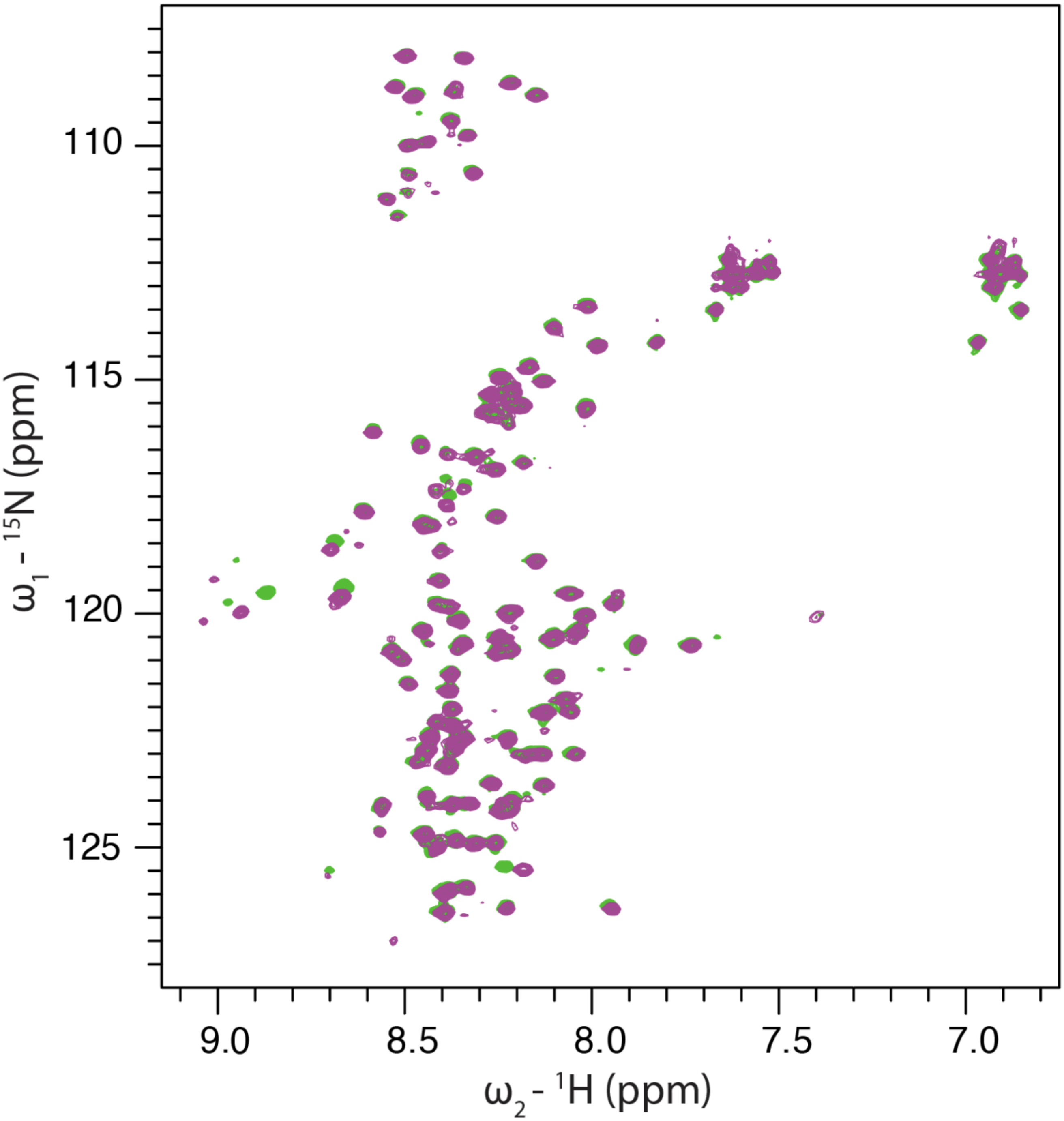

Supplemental Figure 6. Chemical shift conservation of differently labeled pLIC2 samples. Overlay of a previous ^{13}C,^{15}N-labeled pLIC2-C HSQC (green) that was used to assign pLIC2-C residues with a ^{15}N-labeled pLIC2-C HSQC (purple) that was used for Pin1 addition to ensure sufficient phosphorylation. Shifts of phosphorylated residues may also be due to buffer conditions, as a new NMR buffer was made between measurements.

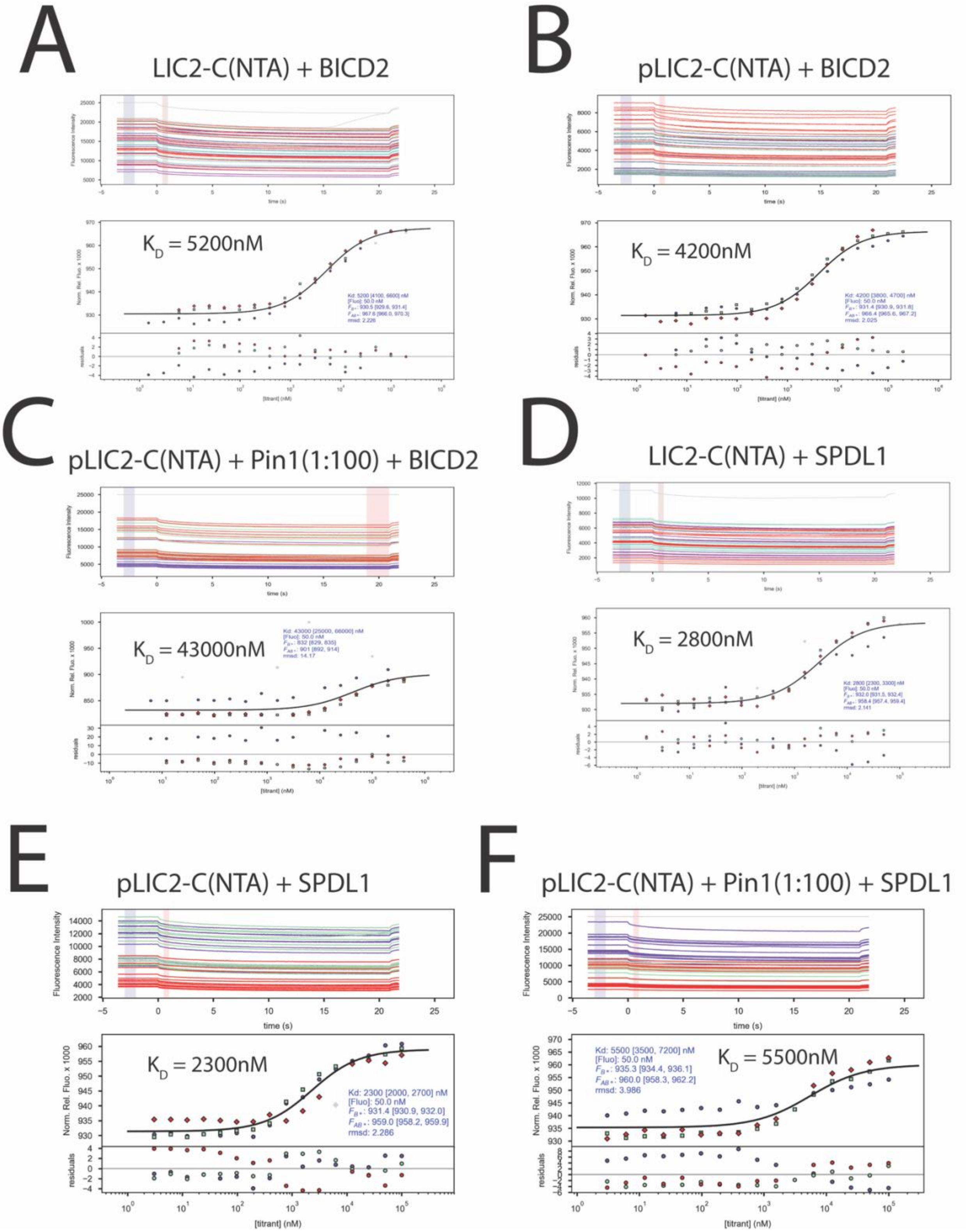
A
LIC2-C(NTA) + BICD2
K_D = 5200nM
B
pLIC2-C(NTA) + BICD2
K_D = 4200nM
C
pLIC2-C(NTA) + Pin1(1:100) + BICD2
K_D = 43000nM
D
LIC2-C(NTA) + SPDL1
K_D = 2800nM
E
pLIC2-C(NTA) + SPDL1
Fluorescence Intensity
time (s)
Norm. Rel. Fluo. x 1000
K_D = 2300nM
Kd: 2300 [2000, 2700] nM
[Fluo]: 50.0 nM
F_B*: 931.4 [930.9, 932.0]
F_AB*: 959.0 [958.2, 959.9]
rmsd: 2.286
residuals
[titrant] (nM)
F
pLIC2-C(NTA) + Pin1(1:100) + SPDL1
Fluorescence Intensity
time (s)
Norm. Rel. Fluo. x 1000
Kd: 5500 [3500, 7200] nM
[Fluo]: 50.0 nM
F_B*: 935.3 [934.4, 936.1]
F_AB*: 960.0 [958.3, 962.2]
rmsd: 3.986
K_D = 5500nM
residuals
[titrant] (nM)

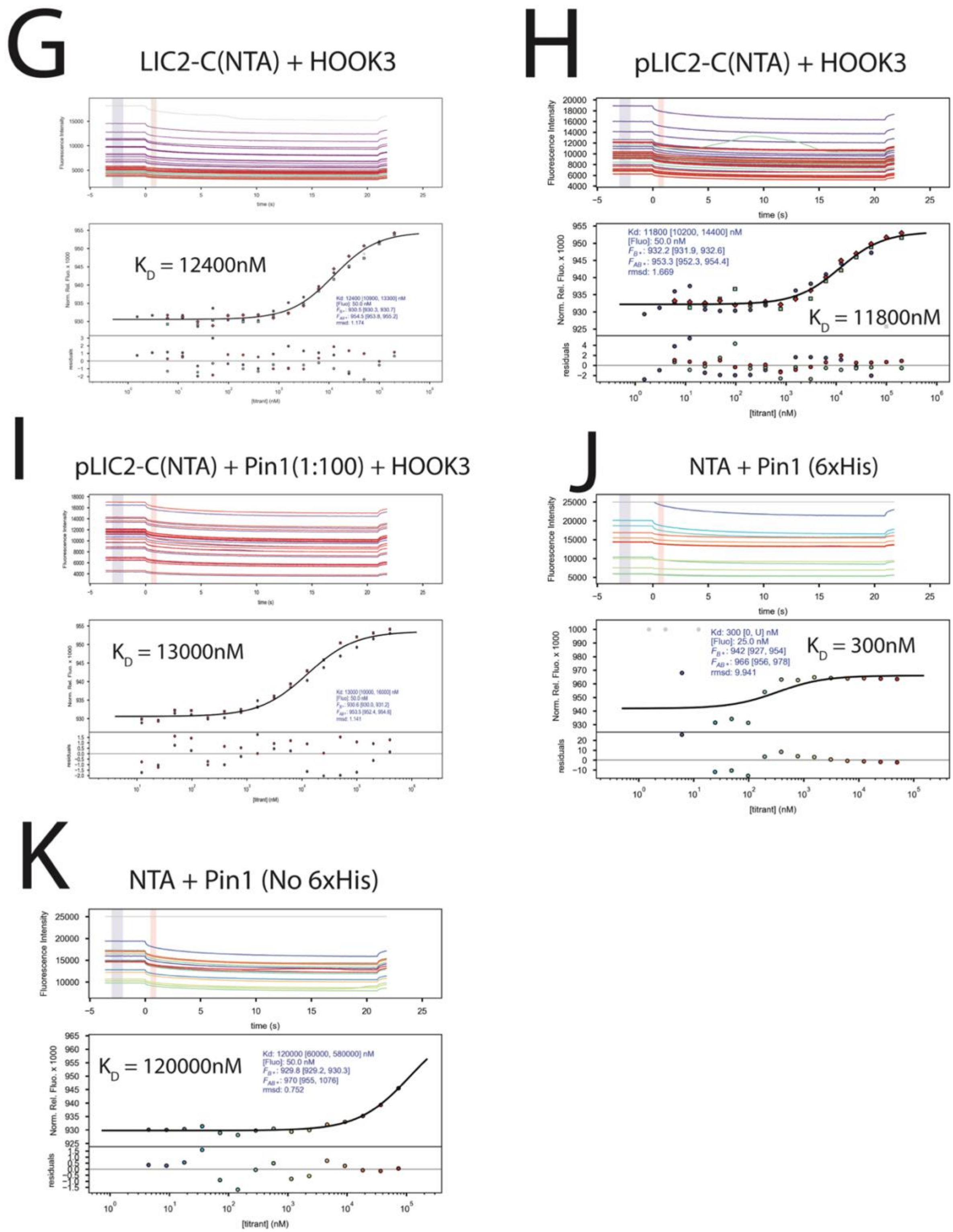

Supplemental Figure 7: MST titration experiments performed to fit response curves and assess binding. A-C) Globally fit MST response curves of NTA-labeled LIC2-C, pLIC2-C and pLIC2-C in the presence of Pin1 (No 6xHis) with BICD2. D-F) Globally fit MST response curves of NTA-labeled LIC2-C, pLIC2-C, and pLIC2-C in the presence of Pin1 (No 6xHis) with SPDL1. G-I) Globally fit MST response curves of NTA-labeled LIC2-C, pLIC2-C, and pLIC2-C in the presence of Pin1 (No 6xHis) with HOOK3. J) NTA-dye titrated with Pin1 (6xHis). K) NTA-dye titrated with Pin1 (No 6xHis).

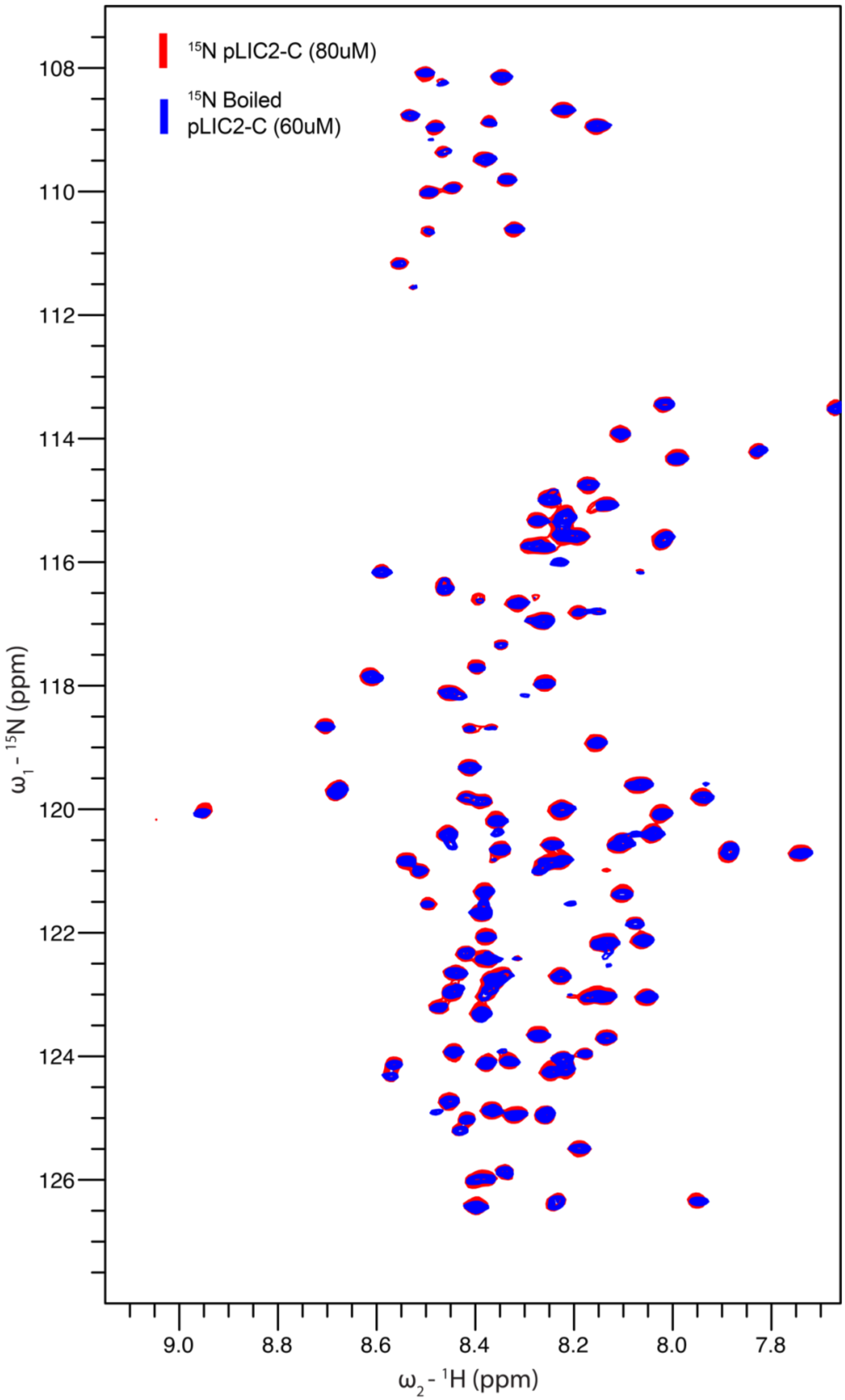

Supplemental Figure 8: Sample integrity and contaminant test after phosphorylation. Overlay of pLIC2-C HSQC (red) and boiled-pLIC2-C HSQC (blue).

References:

(1) Henen, M. A.; Paukovich, N.; Prekeris, R.; Vögeli, B. Solution NMR Backbone Assignment of the C-Terminal Region of Human Dynein Light Intermediate Chain 2 (LIC2-C) Unveils Structural Resemblance with Its Homologue LIC1-C. *Magnetochemistry* **2023**, *9* (7). DOI: 10.3390/magnetochemistry9070166 From NLM.

(2) Holehouse, A.S.; Ahad, J.; Das, R.K.; Pappu, R.V. CIDER: Classification of Intrinsically Disordered Ensemble Regions. *Biophys. J.* **2015**. *108*, 228
